# Ag-CuO Nanozymes Superior to Commercial Silver-Based Dressings: Synergistic Antibacterial Therapy via PTS-Mediated Starvation and Cuproptosis-Like Death

**DOI:** 10.34133/research.1297

**Published:** 2026-06-11

**Authors:** Shuo Zhang, Dongliang Yang, Yunfeng Zheng, Yangyang Liu, Xinyu Zhao, Yutong He, Xu-Lin Chen, Xianwen Wang

**Affiliations:** ^1^ Department of Burns, The First Affiliated Hospital of Anhui Medical University, Hefei 230032, P. R. China.; ^2^ School of Physical and Mathematical Sciences, Nanjing Tech University, Nanjing 211816, P. R. China; ^3^ Department of Orthopedics, First Affiliated Hospital of Bengbu Medical University, Bengbu 233000, P. R. China.; ^4^ Anhui Province Key Laboratory of Occupational Health, Anhui No. 2 Provincial People’s Hospital, Hefei 230041, P. R. China.; ^5^ School of Biomedical Engineering, Anhui Medical University, Hefei 230032, P. R. China.

## Abstract

Burn wounds are prone to infection, and traditional antibiotics cannot be used locally due to bacterial resistance. As a clinical first-line use of silver ion dressings, their use is often limited due to potential toxic side effects and high costs. To overcome these limitations, a bimetallic nanozyme with multiple antibacterial properties was proposed. CuO nanoflowers were first synthesized via a simple liquid-phase method, and then, Ag ions were doped to synthesize Ag-CuO nanozymes. In vitro/in vivo experiments/RNA sequencing revealed that it not only has a variety of enzyme activities, which can accumulate reactive oxygen species (ROS) for sterilization, but can also cooperate with the starvation-cuproptosis-like death cascade for sterilization. The genes regulating the phosphotransferase system were down-regulated by Ag-CuO nanozymes, which reduced the uptake of carbohydrates needed for energy synthesis by bacteria. The tricarboxylic acid cycle signaling pathway was inhibited, and adenosine triphosphate synthesis was reduced, thus inhibiting its own protection against external stimuli. Moreover, the down-regulation of the *lpdA* gene, coupled with bacterial starvation, synergistically led to an increase in Cu^2+^ influx and an increase in Cu^+^ accumulation, thereby amplifying bacterial cuproptosis-like death and biofilm inhibition. The unique ROS-starvation-cuproptosis-like death sterilization method overcomes the limitation that traditional antibiotics are easily tolerated by bacteria. Importantly, its efficacy was verified in a mouse New Zealand rabbit model, which strongly demonstrated its potential for clinical use in the treatment of drug-resistant bacterial infections, as it has excellent antibacterial activity and the ability to promote angiogenesis compared with those of commercial silver-based dressings with the same silver ion content.

## Introduction

Burns refer to damage caused by external factors such as heat (flame, hydrothermal solution, steam, high-temperature solid, etc.), chemical substances (strong acid, strong alkali, etc.), current, radiation, or low temperature (special type of frostbite) [1]. According to the depth and severity of injury, burns can be divided into different grades, which are usually accompanied by local tissue necrosis and inflammatory reactions and can cause systemic pathophysiological changes in severe cases. When burns occur, the natural barrier function of the skin is damaged, necrotic tissue remains, immune function is inhibited, and long-term hospitalization (such as catheter and ventilator use) easily results in bacterial colonization or invasion of the wound, resulting in a complex microbial environment [2]. If treatment is not timely, local infection not only affects wound healing but also causes bacteremia and sepsis, leading to death. Relevant data show that the mortality rate of infected burn patients is twice as high as that of uninfected burn patients [3]. For bacterial infections, antibiotics are still the first choice in the clinic [4]. However, owing to the local use and abuse of antibiotics, the emergence of drug-resistant bacteria such as methicillin-resistant *Staphylococcus aureus* (MRSA) [5,6], vancomycin-resistant *Enterococcus* (VRE) [7,8], and *Pseudomonas aeruginosa* (*P. aeruginosa*) [9] has accelerated. Drug-resistant bacteria markedly increase the difficulty of treatment through complex drug resistance mechanisms such as natural drug resistance, immune escape, drug resistance gene transfer, and biofilm formation [10,11]. Faced with this dilemma, scholars worldwide have attempted to overcome the bottleneck of treatment through various technical paths, such as bacteriophage therapy [12], CRISPR-Cas gene editing technology [13], and photodynamic therapy [14]. However, their therapeutic effects are limited by problems such as poor targeting ability and poor permeability [15,16].

In addition, burn wound infection is usually caused by mixed infection with various pathogens rather than a single strain, and different kinds of bacteria interact. *Enterococcus faecalis* (*E. faecalis*) is a common conditional pathogen in burn wound infection. Although it is not the most common pathogen, it has strong drug resistance and easily results in mixed infections. It can not only increase the release of virulence factors from *Escherichia coli* (*E. coli*) but also transfer drug resistance genes to intraspecies and other strains, making it very difficult to treat [17,18]. The phosphoenolpyruvate (PEP)–phosphotransferase system (PTS) is a complex device in bacteria [19]. This polyprotein phosphorylation chain can recognize extracellular signals (such as sugars) and intracellular signals (such as PEP and nitrogen) and transform them into regulators of target activity through protein–protein interactions [20]. PTS was first found in *E. coli* and has also been found in many other bacteria in recent years. It not only is a sugar transport system that mediates carbohydrate uptake and phosphorylation but also has a very strong regulatory ability, participating in the regulation of central carbon and nitrogen metabolism, regulating metal ion homeostasis, and regulating bacterial virulence, which is closely related to biofilm formation and drug resistance. Therefore, as a group of proteins shared by bacteria, PTS may be effective targets for the treatment of bacterial infection after burn injury.

Silver has long been applied in the clinic because of its superior antibacterial properties, and it can consume antioxidant reserves, which leads to an increase in reactive oxygen species (ROS) in bacteria and replacement with metal ions, resulting in the inhibition of protein activity [21]. However, its wide clinical application is limited by its cytotoxicity, drug resistance risk, local irritation, and inconvenience. Copper-based nanozymes have attracted widespread attention in antibacterial research in recent years because of their good catalytic performance and unique antibacterial mechanism [22,23]. This substance catalyzes the decomposition of H_2_O_2_, yielding ROS, which damage bacterial cellular integrity [24]. Moreover, copper ions can enter bacterial cells through active or passive transport mechanisms and are further reduced to highly active Cu^+^ in cells, thus mediating oxidative damage and metabolic disorders and subsequently inducing cuproptosis-like processes [25,26]. However, cuproptosis-like effects are clearly concentration dependent, and excessive copper ions can cause toxicity to cells and tissues of the body [27]. Therefore, strategies involving multiple antibacterial mechanisms have been proposed, such as the construction of antibacterial platforms, such as copper/molybdenum bimetallic [28], copper/zinc bimetallic [29], and copper/cobalt bimetallic platforms [30]. Some scholars have doped silver and CuO to prepare new nanozymes that show synergistic antibacterial ability. However, these studies are relatively limited by the phenotypes of “destroying membrane/protein and increasing ROS”, and the related mechanisms have not been elucidated [31]. A full study of the antibacterial mechanism will provide a key scientific basis and technological breakthrough for the research and development of new targeted therapeutic drugs [32].

**Fig. 1.**
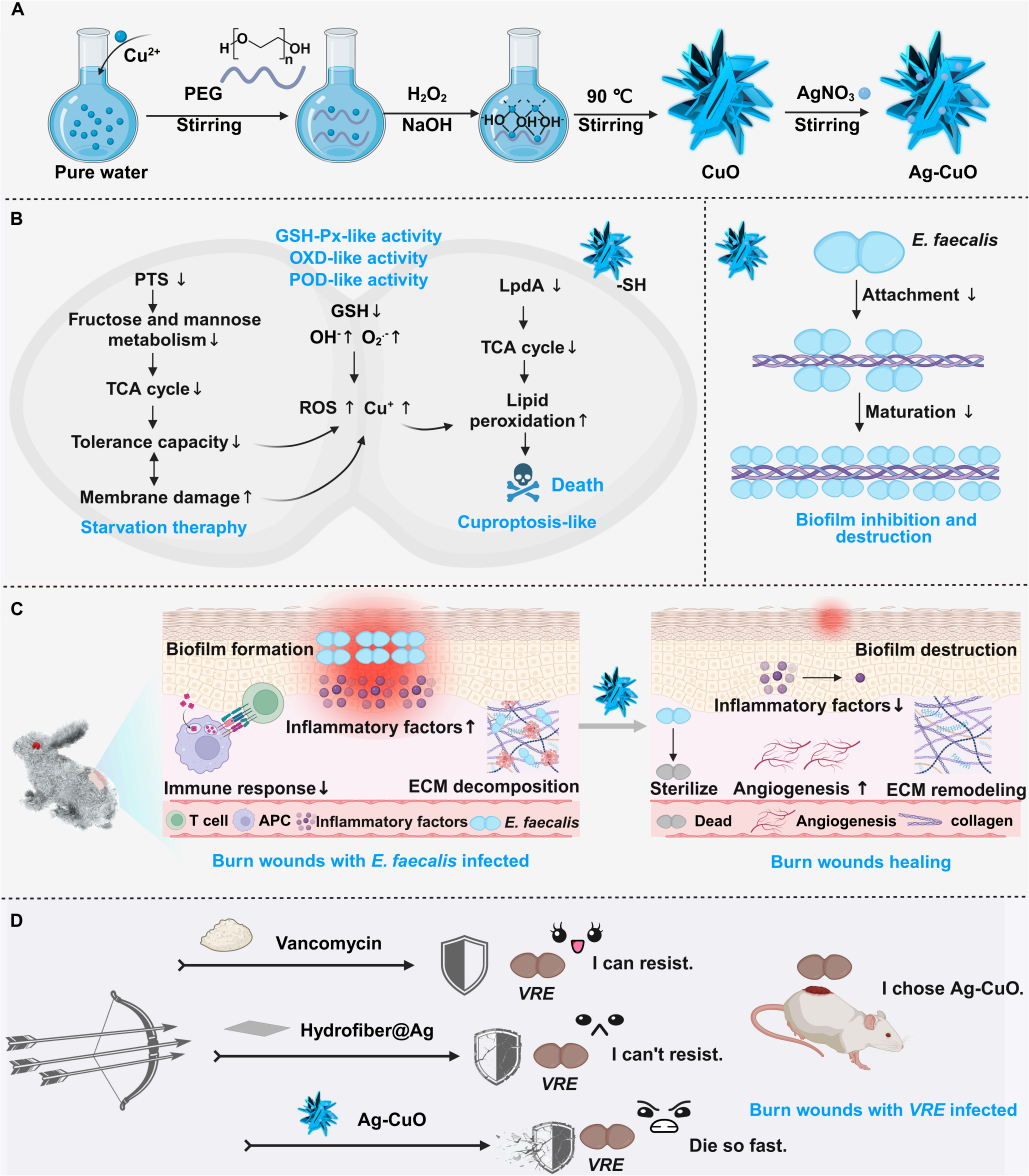
(A) Schematic diagram of the synthesis steps of Ag-CuO nanozymes. (B) Potential mechanism of Ag-CuO nanozyme sterilization and biofilm inhibition. (C) The mechanism by which *E. faecalis* hinders the healing of burn wounds and the mechanism by which Ag-CuO nanozymes promote wound healing. (D) Comparison of the efficacy of Ag-CuO nanozymes with Van and Hydrofiber@Ag in the treatment of burn wounds with VRE infection.

Here, a bimetallic nanozyme with a “ROS-starvation-cuproptosis-like death” antibacterial cascade that promotes wound healing after burn infection was proposed (Fig. 1). Owing to the low bioavailability of silver, CuO nanoflowers wrapped with polyethylene glycol (PEG) were first synthesized via a hydrothermal method and then doped with silver to synthesize bimetallic nanozymes (Ag-CuO). The advantages of the nanozyme are as follows: (a) strong dispersion and uniform structure; in vitro/in vivo experiments/RNA sequencing revealed that (b) Ag-CuO nanozymes have excellent multienzyme activities, which can produce ROS to antibacteria; and (c) Ag-CuO nanozymes can down-regulate (*manX/Y/Z* and *fruA/B/K*) genes to regulate the PTS transferase system and reduce the uptake of carbohydrates by bacteria, resulting in a decrease in adenosine triphosphate (ATP) synthesis, thus reducing tolerance to external stimuli. Moreover, Ag-CuO nanozymes down-regulate the *lpdA* gene, and bacterial starvation reduces its own protective effect, which leads to an increase in Cu^2+^ influx and Cu^+^ accumulation, thus amplifying bacterial cuproptosis-like death and biofilm inhibition. That is, through a starvation-cuproptosis-like death antibacterial cascade, (d) the unique ROS-starvation-cuproptosis-like death sterilization method overcomes the limitation that traditional antibiotics are easily tolerated by bacteria. Importantly, the efficacy gradually verified in a mouse New Zealand rabbit model strongly demonstrated its potential for clinical use in drug-resistant bacterial infections, as it has excellent antibacterial activity and the ability to promote angiogenesis compared with those of commercial silver-based dressings with the same silver ion content. The bimetallic nanozyme (Ag-CuO) introduced in this study can resist bacteria via multiple mechanisms, which provides a new solution for the treatment of drug-resistant bacteria after burn injury.

## Results and Discussion

### Synthesis and characterization of Ag-CuO nanozymes

**Fig. 2.**
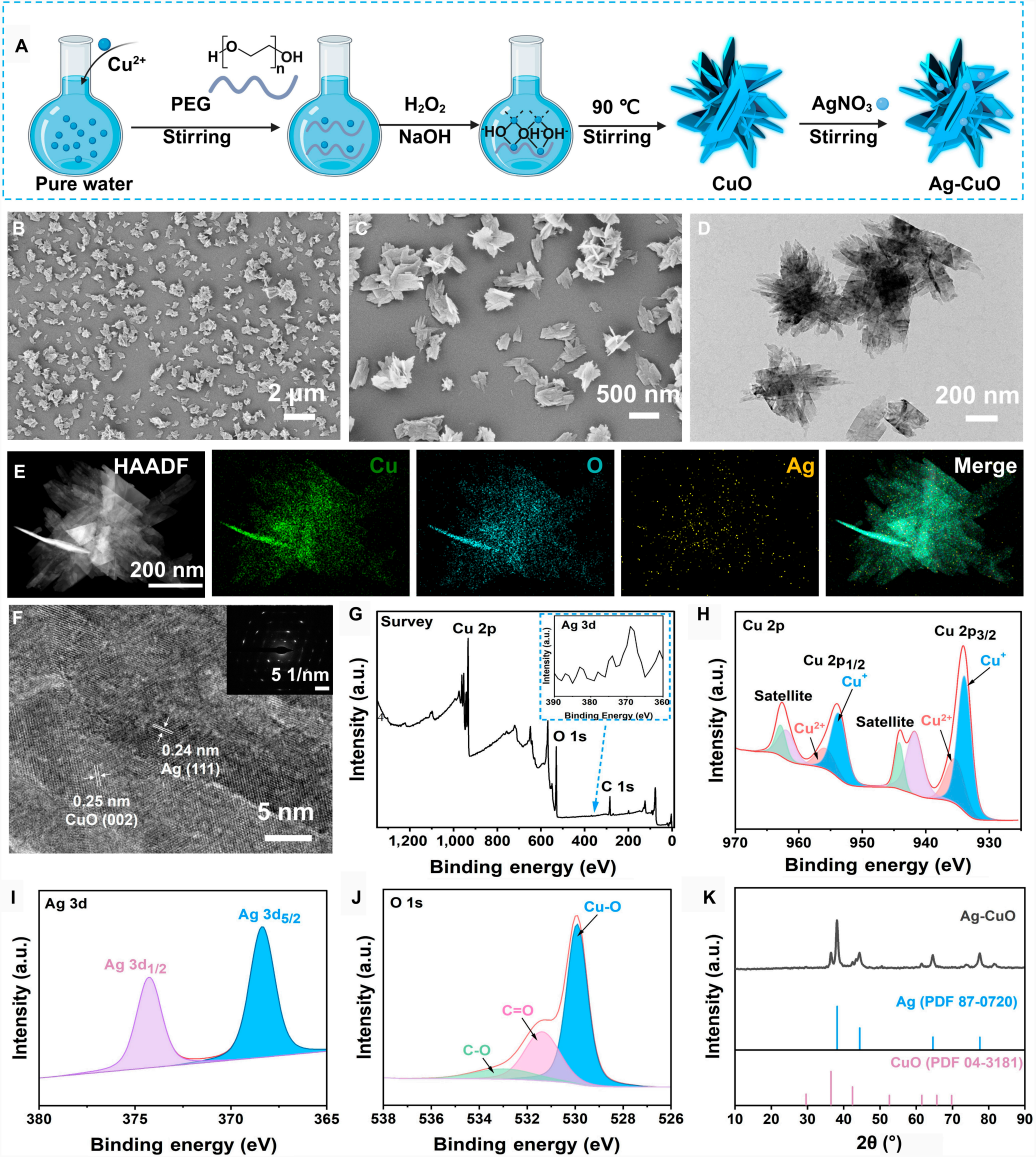
Synthesis and characterization of Ag-CuO nanozymes. (A) Mechanistic diagram of synthesis. (B and C) SEM images of the Ag-CuO nanozymes. (D) Transmission electron microscopy (TEM) images of the Ag-CuO nanozymes. (E) HAADF images and elemental mapping images of the Ag-CuO nanozymes. (F) HRTEM image and selected area electron diffraction image of the Ag-CuO nanozymes. (G to J) X-ray photoelectron spectroscopy (XPS) spectra of the Ag-CuO nanozymes. (K) X-ray diffraction (XRD) patterns of the Ag-CuO nanozymes.

The synthesis method was improved on the basis of previous studies [33]. CuO nanoflowers were synthesized via a simple liquid-phase method, AgNO_3_ solution was then added, and Ag-CuO nanozymes were finally successfully prepared (Fig. 2A). The morphology of the Ag-CuO nanozymes was observed by scanning electron microscopy (SEM) and transmission electron microscopy (TEM). The diameter of the Ag-CuO nanozymes is approximately 460 nm, their size is uniform, and they have a flower-like morphology. The petals are composed of nanosheets (Fig. 2B to D). The elemental mapping images revealed that the Ag-CuO nanozymes were composed of Cu, O, and Ag and that Ag was uniformly loaded on the CuO nanoflowers (Fig. 2E). The energy-dispersive x-ray spectroscopy (EDS) spectra also show that the Ag-CuO nanozymes contain Cu, O, Ag, and other elements (Fig. S1). Furthermore, Ag-CuO nanozymes were observed via high-resolution TEM (HRTEM). The measured lattice spacing of 0.24 nm matches well with the plane of Ag (111), and the measured lattice spacing of 0.25 nm matches well with the plane of the monoclinic CuO phase (002). The selected area electron diffraction pattern also shows that the Ag-CuO nanozymes are polycrystalline (Fig. 2F and Fig. S2). The formation of Ag-CuO nanozymes was characterized by x-ray photoelectron spectroscopy (XPS). The results revealed obvious Cu 2p, Ag 3d, O 1 s, and C 1 s peaks in Ag-CuO nanozymes (Fig. 2G). The Cu 2p spectra show 4 peaks. The main peaks at 934.1 and 954.1 eV are attributed to 0Cu 2p_3/2_ and Cu 2p_1/2_, respectively. There is a band gap of 20 eV between the 2 peaks, which is consistent with previous studies [31]. The other 2 peaks are satellite peaks (Fig. 2H). Ag 3d has 2 peaks, one of which is Ag 3d_5/2_ at 368.3 eV, and the other is Ag 3d_1/2_ at 374.3 eV. There is a difference of 6 eV between the 2 peaks, which may be due to spin–orbit coupling (Fig. 2I). The O 1 s peak at 529.7 eV was attributed to the binding site between the Cu and O metals (Fig. 2J). Moreover, the crystal structures of the Ag-CuO nanozymes were analyzed via powder x-ray diffraction (XRD). The results show that the Ag-CuO nanozymes contain face-centered cubic (fcc) Ag (PDF No. 87-0720) and monoclinic CuO planes (PDF No. 04-3181) (Fig. 2K). The inductively coupled plasma optical emission spectrometry (ICP-OES) results revealed that the content of Cu was 79.56 WT% and that of Ag was 0.46 WT%. Through the above characterization techniques and results, Ag-CuO nanozymes were successfully synthesized.

### Multienzyme-like activities and ion release of Ag-CuO nanozymes

**Fig. 3.**
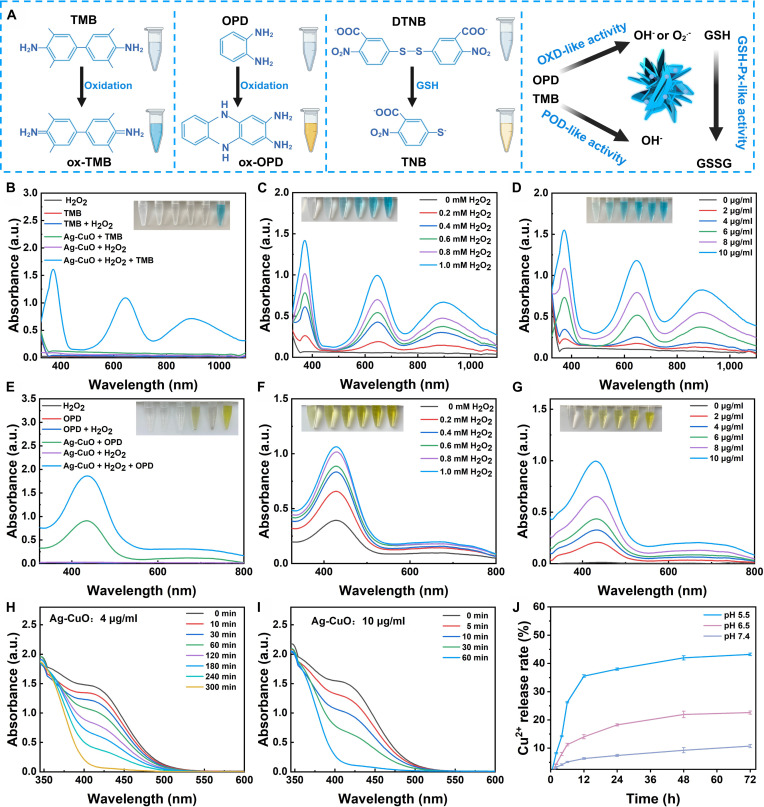
Multienzyme-like activities and ion release of Ag-CuO nanozymes. (A) Schematic diagram of the multienzyme-like activities. (B) UV–vis spectra of TMB in different groups (H_2_O_2_, TMB, TMB + H_2_O_2_, Ag-CuO + TMB, Ag-CuO + H_2_O_2_, and Ag-CuO + H_2_O_2_ + TMB). (C) UV–vis spectra of TMB in different concentrations of H_2_O_2_. (D) UV–vis spectra of TMB in different concentrations of Ag-CuO nanozymes. (E) UV–vis spectra of the OPDs in different groups. (F) UV–vis spectra of OPD in different concentrations of H_2_O_2_. (G) UV–vis spectra of OPD with different concentrations of Ag-CuO nanozymes. (H and I) The time dependence of GSH depletion in Ag-CuO nanoflowers at concentrations of 4 and 10 μg/ml was determined with a DTNB probe. (J) The percentage of Cu^2+^ dissociated by the Ag-CuO nanozymes in different pH solutions. The error bars represent the means ± SDs (*n* = 3).

The multienzyme activities of copper-based nanozymes have been verified [34,35]. To determine whether Ag doping affects the multienzyme activities of CuO, 3 probes, TMB (3,3′,5,5′-tetramethylbenzidine probe), OPD (1,2-bbenzylenediamine), and 5,5′-dithiobis (2-nitrobenzoic acid) (DTNB), were used to detect the multienzyme activities (Fig. 3A). Moreover, the release ability of the metal ions from the Ag-CuO nanozymes at different pH values was detected via ICP. The antibacterial mechanism of Ag-CuO nanozymes can be better explained by fully characterizing the various enzyme activities of Ag-CuO nanozymes.

The color reaction of TMB oxidation is mainly due to the presence of benzidine, which contains 2 easily oxidized amino groups and can be oxidized into blue products by single-electron and 2-electron pathways. The oxidation process of benzidine is divided into 2 steps. The 371- and 652-nm bands are charge transfer complexes composed of diamine (TMB) as the donor and diimine ion (TMB^2+^) as the acceptor. Therefore, if Ag-CuO nanozymes have peroxidase activity (POD), they will catalyze hydrogen peroxide to form OH· under acidic conditions (pH = 5.5), and the reaction between OH· and TMB will produce blue ox-TMB. By setting different grouping control variables, we finally verified that Ag-CuO nanozymes can catalyze the oxidation of hydrogen to OH· under acidic conditions (Fig. 3B). With increasing hydrogen peroxide or Ag-CuO concentration, the absorption peak at 652 nm gradually increased (Fig. 3C and D), which indicated that Ag-CuO nanozymes have superior POD-like activity. Electron spin resonance spectroscopy with 5,5-dimethyl-1-pyrroline N-oxide (DMPO) as the spin trap was performed to confirm the generation of hydroxyl radicals (OH·) in the Ag-CuO nanozyme + H_2_O_2_ system. As shown in Fig. S3, a characteristic multiline EPR signal was detected in the Ag-CuO + H_2_O_2_ group, which was primarily assigned to the DMPO-OH adduct (the specific spin adduct of OH·), with a minor contribution from the DMPO-OOH adduct (the spin adduct of superoxide anion, O_2_·^−^). These results directly demonstrated that Ag-CuO nanozymes could efficiently catalyze the decomposition of H_2_O_2_ to generate OH·, which served as the core bactericidal ROS in the synergistic antibacterial cascade.

OPD is a peroxidase substrate. After being oxidized, it will produce orange–brown ox-OPD, which can be read by a spectrophotometer at 435 nm. We also tested different concentrations of hydrogen peroxide or Ag-CuO nanozymes to verify that Ag-CuO nanozymes have superior POD-like activity (Fig. 3F and G). Interestingly, when different grouping control variables were used, even without the addition of hydrogen peroxide, the Ag-CuO nanozymes themselves would oxidize OPD to orange–brown ox-OPD (Fig. 3E), indicating that they also had OXD-like activity.

As a tripeptide, glutathione has a γ-amide bond and a sulfhydryl group, occurring in both reduced (GSH) and oxidized (GSSG) forms [36]. GSH has antioxidant effects. To detect whether Ag-CuO nanozymes can consume glutathione and reduce the removal of ROS by bacteria, a DTNB probe was used. DTNB reacts with GSH to produce orange–yellow TNB, which can be read by a spectrophotometer at 412 nm. The results show that GSH is gradually consumed with increasing time, and the time consumption becomes shorter with increasing Ag-CuO nanozyme concentration. When the concentration of Ag-CuO nanozymes was 10 μg/ml, it was depleted in only 60 min, which indicates that Ag-CuO nanozymes have superior glutathione-like peroxidase (GSH-Px-like) activity (Fig. 3H and I).

The activity of metal nanozymes is closely related to the concentration of metal ions that are dissociated. Three different pH values were used to detect the release ability of the Ag-CuO nanozyme metal ions. Compared with the other 2 pH values, the Ag-CuO nanozymes have the best ability to release Cu and Ag ions at pH = 5.5. At pH = 5.5, approximately 37% of the Cu ions and 12% of the Ag ions were released from the Ag-CuO nanozymes at 12 h (Fig. 3J and Fig. S4).

The above experimental results show that Ag-CuO nanozymes have excellent metal ion release ability at pH 5.5. It not only has OXD-like and POD-like activities to produce ROS but also has GSH-Px-like activity to reduce ROS clearance, resulting in the bidirectional accumulation of ROS, and has superior antibacterial potential.

### Broad-spectrum antibacterial activity of Ag-CuO nanozymes in vitro

The multienzyme activity of the Ag-CuO nanozymes was fully verified in vitro. To determine whether Ag-CuO nanozymes have superior bactericidal functions, plate tests and bacterial growth curves were used for verification. Although hydrogen peroxide is a commonly used disinfectant for treating wound infection in the clinic, when its concentration is high, it often causes oxidative damage to normal cells and affects wound healing [37]. On the one hand, a low dose of hydrogen peroxide (100 μM) can be used as an exogenous oxide donor; on the other hand, it will not cause damage to cells at a very low concentration, thus ensuring safety. The infection of burn wounds is often not caused by a single strain [38], so 2 gram-positive bacteria, MRSA and *E. faecalis*, and 2 gram-negative bacteria, *P. aeruginosa* and *E. coli*, were selected to test the antibacterial activity of the Ag-CuO nanozymes.

**Fig. 4.**
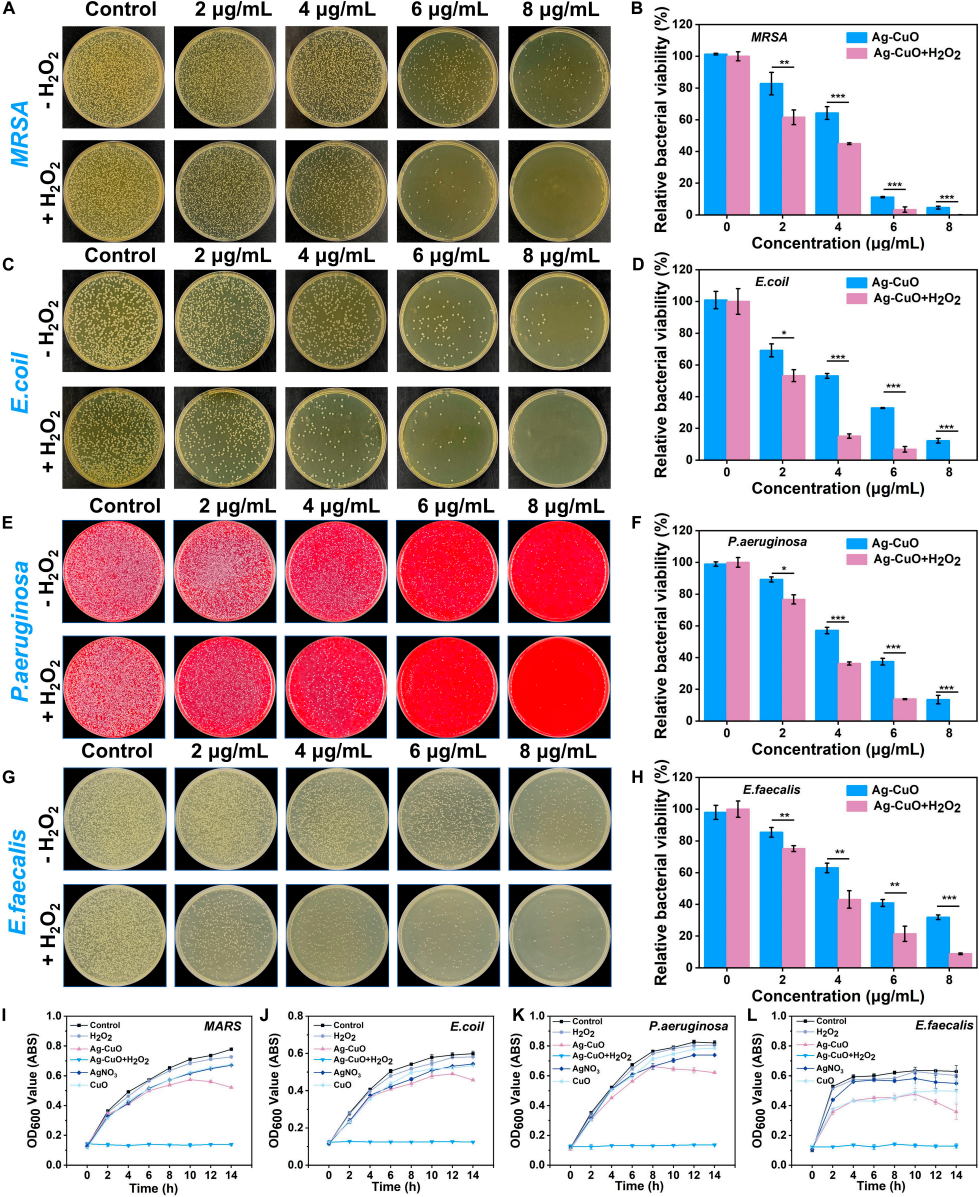
Broad-spectrum antibacterial activity of Ag-CuO nanozymes in vitro. (A and B) Bacterial plate photos and survival rates of MRSA treated with different concentrations of Ag-CuO nanozymes. (C and D) Bacterial plate photos and survival rates of *E. coli* treated with different concentrations of Ag-CuO nanozymes. (E and F) Bacterial plate photos and survival rates of *P. aeruginosa* treated with different concentrations of Ag-CuO nanozymes. (G and H) Bacterial plate photos and survival rates of *E. faecalis* treated with different concentrations of Ag-CuO nanozymes. (I to L) Growth curves of MRSA*, E. coli, P. aeruginosa,* and *E. faecalis* in the different groups. The error bars represent the means ± SDs (*n* = 3).

The results of the plate test revealed that the Ag-CuO nanozymes had antibacterial activity against 4 kinds of bacteria (Fig. 4A, C, E, and G). Compared with the control, exogenous hydrogen peroxide (100 μM) alone had no effect on the bacteria. In the group without hydrogen peroxide, the number of bacteria decreased gradually with increasing Ag-CuO nanozyme concentration, which was accompanied by a concentration dependence. However, after the addition of exogenous hydrogen peroxide, the bactericidal efficacy of the Ag-CuO group at the same concentration was markedly better than that of the group without hydrogen peroxide, which may be related to the POD-like activity of the Ag-CuO nanozymes (Fig. 4B, D, F, and H).

The optimum concentration of antibacterial agent was 8 μg/ml according to the plate test, so the growth inhibition curves of the 4 kinds of bacteria were generated by setting different groups. The results also revealed that the addition of exogenous hydrogen peroxide (100 μM) alone had no effect on bacteria. Compared with AgNO_3_ or CuO alone, the Ag-CuO group presented better antibacterial effects, but after exogenous hydrogen peroxide was added, the effect was qualitatively improved (Fig. 4I to L). The above experimental results show that Ag-CuO nanozymes have excellent broad-spectrum antibacterial properties in vitro and superior prospects in the treatment of burn-infected wounds.

### Evaluation of the killing effect of Ag-CuO nanozymes on *E. faecalis* in vitro

Burn wound infection is usually caused by mixed infection with many pathogens, and there are interactions between different kinds of bacteria [39]. Although *E. faecalis* is not the most common pathogen, it can increase the release of virulence factors and transmit drug resistance genes to other bacteria, and its cell wall is thick, resulting in inherent resistance to many antibiotics. If it progresses to VRE, it threatens the life of the body [40]. Therefore, this study focused on the antibacterial activity of *E. faecalis*. The killing effect of Ag-CuO nanozymes on *E. faecalis* was evaluated via plate tests, live and dead fluorescence staining, SEM, ROS fluorescence staining, flow cytometry, and lipid peroxidation (LPO) fluorescence staining. In previous work, different concentrations of Ag-CuO nanozymes were used for plate tests, so the best concentration of 8 μg/ml was used for the following experiments, and the effects of the Ag-CuO nanozymes were better than those of AgNO_3_ or CuO according to the bacterial growth curve. To reduce the experimental cost, the experimental groups used were the control, H_2_O_2_, Ag-CuO, and Ag-CuO + H_2_O_2_ groups.

**Fig. 5.**
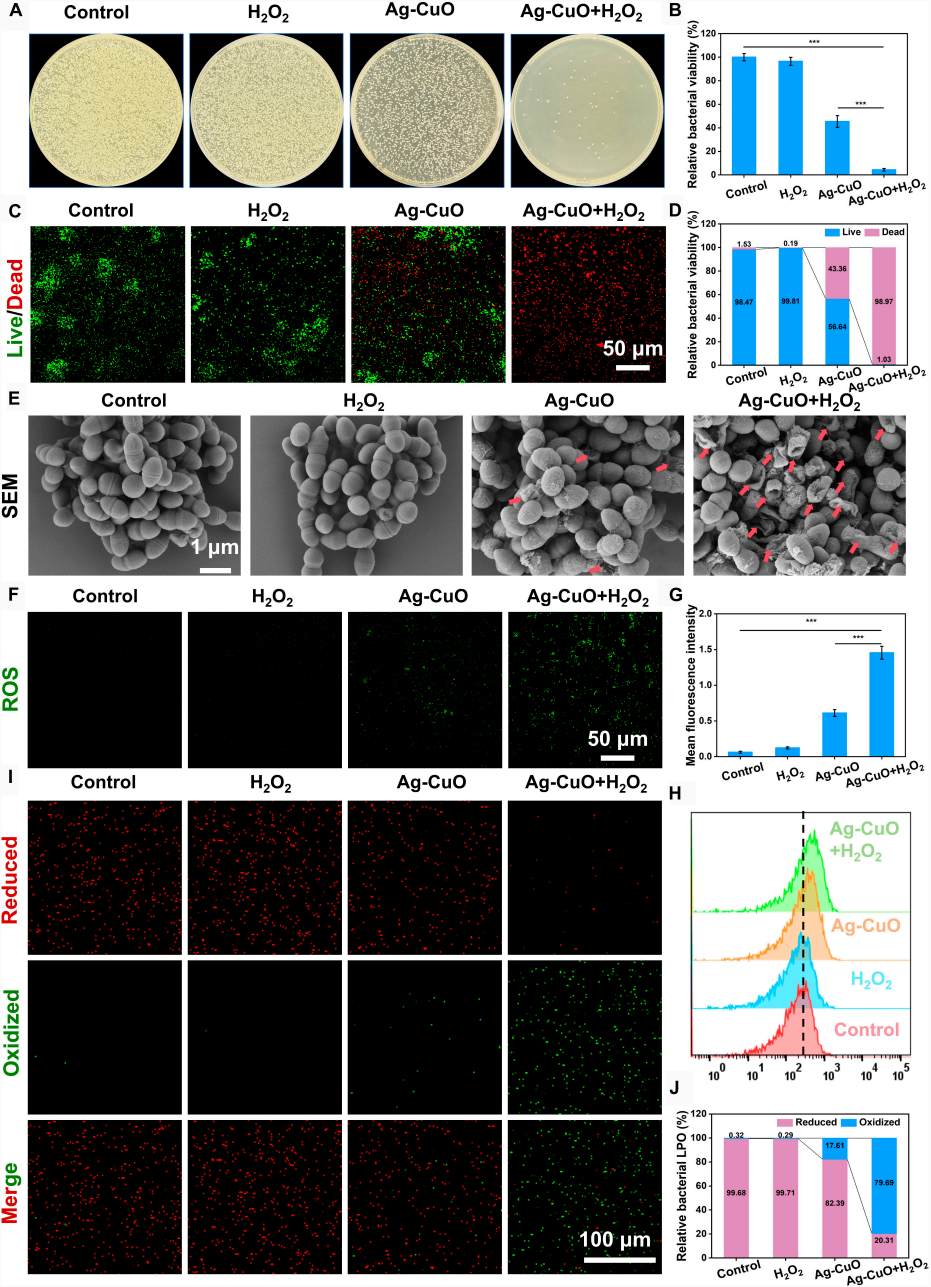
Evaluation of the killing effect of Ag-CuO nanozymes on *E. faecalis* in vitro. (A and B) Bacterial plate photos and survival rates of *E. faecalis* in different groups. (C and D) Fluorescence images of bacteria with N01/PI (Live/Dead staining) under different treatments. (E) SEM images of live/dead bacteria under different treatments. (F and G) Fluorescence images of ROS after different treatments. (H) ROS flow cytometry assay. (I and J) Fluorescence images of lipid peroxidation (LPO) under different treatments. The error bars represent the means ± SDs (*n* = 3).

The results of the plate test revealed that Ag-CuO nanozymes alone showed germicidal ability, but at the same concentration, the Ag-CuO + H_2_O_2_ group had the best germicidal effect (Fig. 5A and B), which corresponded to the above results. A live bacteria/dead bacteria staining kit (N01/PI) can reliably stain and quantitatively distinguish live bacteria from dead bacteria within a few minutes via differences in the integrity of the bacterial cell membrane. The living bacteria staining probe (N01) is a green fluorescent-labeled living bacteria probe that can penetrate the plasma membrane with the complete structure of living bacteria. The dead bacteria staining probe with a damaged membrane is PI, which is a red fluorescent nucleic acid staining agent that can penetrate only dead bacteria with damaged membranes. Live/dead fluorescence staining revealed a large amount of green fluorescence and red fluorescence in the Ag-CuO group via confocal microscopy, which indicated that Ag-CuO nanozymes could dissociate Ag^+^ and Cu^2+^ and destroy the cell membrane of bacteria in a weakly acidic environment; however, the effect was limited, and the survival rate of bacteria in the Ag-CuO nanozymes group was 56.64% that of the control group. After H_2_O_2_ was added, almost all the bacteria emitted red fluorescence under a confocal microscope, and the survival rate of the bacteria in the H_2_O_2_-treated group was only 1.03% of that in the control group. We speculated that Ag-CuO nanozymes could catalyze low concentrations of H_2_O_2_ and produce more OH· to kill bacteria in weakly acidic bacterial environments (Fig. 5C and D). To further determine how the Ag-CuO nanozymes interact with bacteria directly, SEM was used to determine that the Ag-CuO nanozymes tightly adhered to the surface of the bacterial cell membrane and destroyed the bacterial cell membrane and that damage to the bacterial cell membrane clearly increased after the addition of H_2_O_2_ (Fig. 5E). 2′,7′-dichlorodihydrofluorescein diacetate (DCFH-DA) exhibits no fluorescence and is membrane permeable. Upon their cellular uptake, esterases metabolize them into DCFH. ROS in cells can oxidize nonfluorescent DCFH to produce green DCF. To validate the hypothesis that Ag-CuO nanozymes can catalyze the production of more OH· at low concentrations of H_2_O_2_ in weakly acidic bacteria, we evaluated the ROS production ability via ROS fluorescence staining and flow cytometry. The results of confocal microscopy revealed that the Ag-CuO nanozymes produced sparse green light, whereas the Ag-CuO + H_2_O_2_ group produced more green light, which was approximately 1.4 times greater than that of the control group (Fig. 5F and G), and the results of the flow experiments were consistent with these findings (Fig. 5H). LPO is a key change in many pathological states [41]. Under conditions of diminished bacterial antioxidant capacity, peroxidative damage occurs to phospholipids containing unsaturated long-chain fatty acids in cellular and organelle membranes, ultimately leading to membrane disruption. To further evaluate the effects of Ag-CuO nanozymes on LPO damage to bacteria, we performed BODIPY 581/591 C11 fluorescence staining. The fluorescence confocal microscopy results revealed that the control and H_2_O_2_ groups were red, which is a reduced product, whereas the Ag-CuO nanozymes group produced sparse green light, and almost all the Ag-CuO + H_2_O_2_ groups produced green lipid peroxides, which were approximately 79.69% of those of the control group (Fig. 5I and J). The results indicated that the antioxidant capacity of the Ag-CuO + H_2_O_2_ treatment decreased markedly, which may be related to the GSH-Px-like activity of the Ag-CuO nanozymes.

The above experimental results show that Ag-CuO nanozymes can adhere to the bacterial membrane and destroy the bacterial membrane structure through ion release and the generation of ROS through the activities of multiple enzymes. Moreover, GSH consumption promotes the collapse of the bacterial antioxidant system, resulting in bacterial death.

### The inhibition and destruction of *E. faecalis* biofilms by Ag-CuO nanozyme treatment

The killing effect of Ag-CuO nanozymes on *E. faecalis* has been verified, but the bacteria present in burn wounds are not single, and *E. faecalis* often exists in the form of biofilms [42]. The formation of biofilms can be roughly divided into the following stages: initial adhesion, stable adhesion, early maturity, maturity, and separation and diffusion. After the formation of biofilms, the drug resistance of bacteria markedly improves, which leads to difficulty in curing and prolonging infection and further aggravates the economic and psychological burdens of patients [43]. Because the characteristics of biofilms cause great problems in clinical treatment, targeted therapy for biofilm infection is particularly important [44]. In this study, we used crystal violet staining, confocal fluorescence staining, and SEM to verify the effects of Ag-CuO nanozymes on the inhibition or destruction of biofilm formation.

**Fig. 6.**
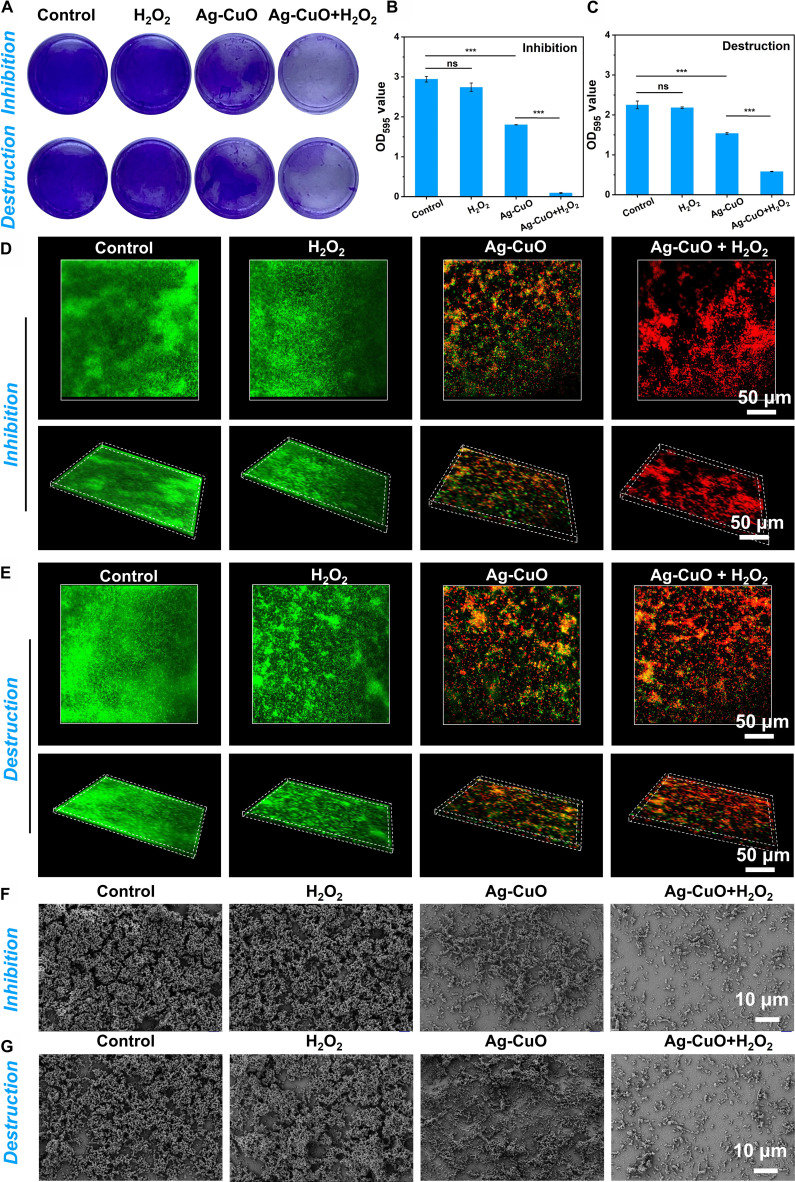
The inhibition and destruction of biofilms by Ag-CuO nanozyme treatment. (A to C) Crystal violet-stained images of *E. faecalis* biofilms subjected to different treatments. (D and E) Fluorescence images of live/dead bacterial biofilms subjected to different treatments. (F and G) Scanning electron microscopy (SEM) images of live/dead bacterial biofilms subjected to different treatments. The error bars represent the means ± SDs (*n* = 3).

Crystal violet staining revealed that Ag-CuO nanozymes alone inhibited and disrupted the formation of biofilms to some extent, whereas H_2_O_2_ addition markedly enhanced the inhibition and destruction of biofilms. Interestingly, we found that after biofilm formation, when the same concentration of drug was used, the effect of destruction was significantly less pronounced than the effect of inhibition, which corresponds to the clinical resistance of bacteria after biofilm formation (Fig. 6A to C). Similarly, we used confocal fluorescence staining to observe the planar and 3-dimensional structure of the biofilm, and the results were consistent with those of crystal violet staining (Fig. 6D and E). The structure of the biofilm was further observed via SEM. The results revealed that the biofilms of the control and H_2_O_2_ groups were very compact, and the bacteria presented obvious adhesion proteins and hyphae. In the Ag-CuO + H_2_O_2_ group, obvious biofilm destruction and residual damaged bacteria were observed (Fig. 6F). On the basis of the above results, Ag-CuO nanozymes have superior effects on both the inhibition of biofilm formation and the destruction of *E. faecalis* biofilms and have good potential for burn-infected wounds.

### Mechanistic hypothesis that *E. faecalis* is killed by Ag-CuO nanozymes

**Fig. 7.**
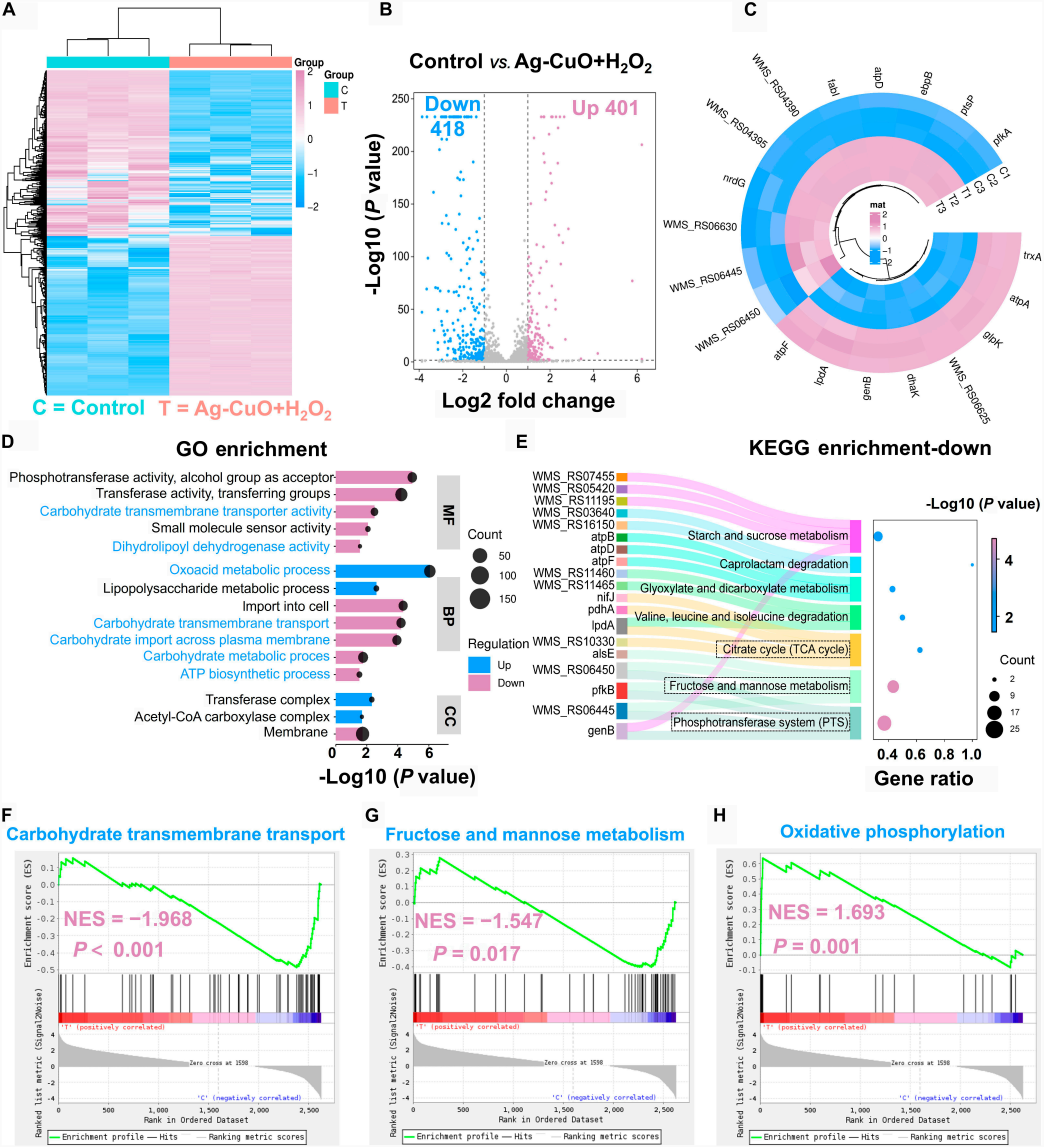
RNA sequencing of *E. faecalis* treated with the control or Ag-CuO + H_2_O_2_ (*n* = 3). (A) Heatmap of significantly down-regulated and up-regulated genes after Ag-CuO + H_2_O_2_ treatment. (B) Volcano maps of genes after Ag-CuO + H_2_O_2_ treatment. (C) Enrichment heatmap of several marker genes. (D) Gene Ontology (GO) enrichment-All. (E) Kyoto Encyclopedia of Genes and Genomes (KEGG) enrichment-down after Ag-CuO + H_2_O_2_ treatment. (F to H) Several gene sets with significant differences after GSEA.

A full study of antibacterial mechanisms will provide a key scientific basis and technological breakthrough for the research and development of new targeted therapeutic drugs [45]. Therefore, we analyzed the transcriptome sequences of the control group and the Ag-CuO + H_2_O_2_ group to further explore the killing mechanism of Ag-CuO nanozymes on *E. faecalis*. Differentially expressed genes (DEGs) thermography revealed that there was a markedly difference in gene set enrichment between the 2 groups (Fig. 7A). The results of the volcano map revealed that 418 genes were down-regulated and that 401 genes were up-regulated in *E. faecalis* treated with Ag-CuO + H_2_O_2_ (Fig. 7B). The circular thermogram revealed representative genes with obvious differences, such as the *ptsP, atpF, atpA, pfkA*, and *lpdA* genes (Fig. 7C). Gene Ontology (GO) enrichment analysis revealed that the DEGs were enriched mainly in carbohydrate transmembrane transporter activity, lipopolysaccharide metabolic process, carbohydrate transmembrane transport, ATP biosynthesis process, acetyl-CoA carboxylase complex, and other pathways (Fig. 7D). Kyoto Encyclopedia of Genes and Genomes (KEGG) enrichment analysis revealed that the down-regulated DEGs were enriched mainly in the PTS, fructose, and mannose metabolism and tricarboxylic acid (TCA) cycle pathways, accompanied by the down-regulation of key genes such as *manX/Y/Z*, *fruA/B/K*, and *lpdA* (Fig. 7E). Gene set enrichment analysis (GSEA) revealed that after Ag-CuO + H_2_O_2_ treatment, carbohydrate transmembrane transport and fructose and mannose metabolism pathways were down-regulated, whereas the oxidative phosphorylation pathway was up-regulated (Fig. 7F to H).

**Fig. 8.**
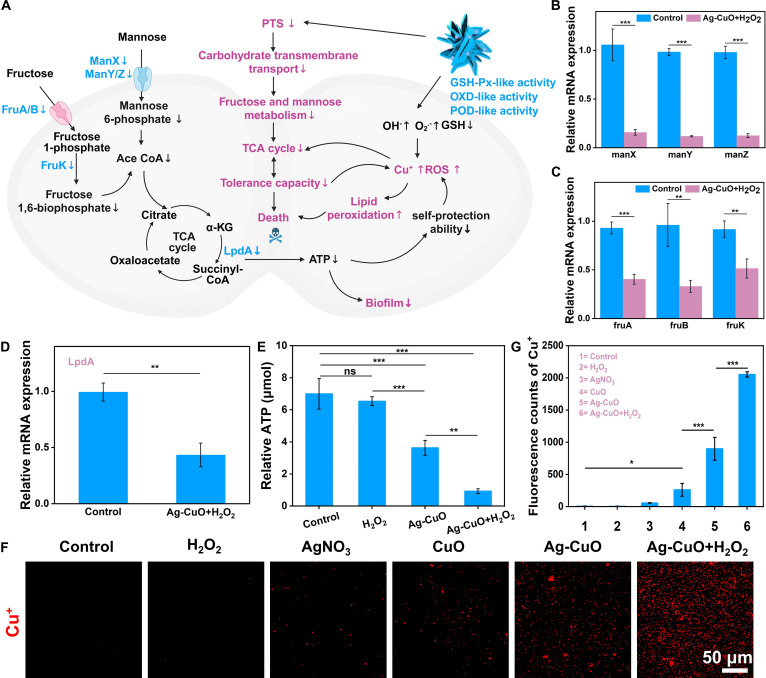
Mechanistic hypothesis that *E. faecalis* is killed by Ag-CuO nanozymes. (A) Mechanistic map of *E. faecalis* killed by Ag-CuO nanozymes. (B and C) RT-qPCR results of PTS-related genes. (D) RT-qPCR results of cuproptosis-like death-related genes. (E) Relative ATP content of *E. faecalis* under different treatments. (F and G) Relative Cu^+^ content of *E. faecalis* under different treatments. The error bars represent the means ± SDs (*n* = 3).

After the analysis of the above transcriptome sequencing results, we preliminarily proposed that the killing mechanism of Ag-CuO nanozymes against *E. faecalis is* as follows: Ag-CuO nanozymes not only have various enzyme activities to accumulate ROS for sterilization but can also cooperate with the antibacterial activity of the starvation-cuproptosis-like death cascade (Fig. 8A).

First, the POD-like, OXD-like, and GSH-Px-like activities of the Ag-CuO nanozymes were verified via various probe experiments and confocal fluorescence staining. It can produce ROS and reduce ROS clearance, thus destroying the bacterial cell membrane and causing LPO damage.

Second, PTS exists in bacteria, fungi, and some archaea, and the basic composition of PTS in the studied species is similar. PTS, which is the key system of cell growth and biofilm formation and can mediate virulence regulation and the stress response, mediates glucose transport and phosphorylation. Interestingly, microorganisms have acquired a self-regulatory mechanism in the evolutionary process to adapt to survive. For example, as long as glucose, fructose or sucrose exist in large quantities in the growth medium, it inhibits the synthesis of enzymes necessary for the transportation and metabolism of nonpreferred carbon sources. This phenomenon is called carbon catabolite inhibition [46]. Therefore, the fructose and mannose metabolism pathways are the preferred carbon sources for *E. faecalis*. After treatment with Ag-CuO + H_2_O_2_, the decrease in genes related to this pathway affected carbon uptake, ATP synthesis, and tolerance to external stimuli. In other words, Ag-CuO nanozymes down-regulate the PTS regulated by *manX/Y/Z* and *fruA/B/K* genes, reduce the carbohydrate intake required for energy synthesis, down-regulate the TCA cycle, and reduce ATP synthesis, thus inhibiting their own protection against external stimuli. This process is called starvation antibacterial activity [47,48]. RT-qPCR was used to validate the *manX/Y/Z* and *fruA/B/K* gene pairs (Fig. 8B and C), and ATP synthesis decreased after Ag-CuO treatment and was more obviously inhibited after the addition of exogenous H_2_O_2_ (Fig. 8E).

Finally, owing to the destruction of the bacterial cell membrane by metal ions released by Ag-CuO nanozymes, especially the interaction between Ag ions and -SH groups on the membrane [49] and the inability of bacterial starvation to resist external stimuli, Cu^2+^ influx and Cu^+^ accumulation increased, thus amplifying the cuproptosis-like death characteristics of bacteria. *LpdA* is a key protein in the TCA cycle that serves as the E3 component of the α-ketoglutarate dehydrogenase complex and connects to the mitochondrial electron transport chain [50]. Mutation of the *lpdA* gene causes excessive accumulation of Cu^+^ in cells, which induces copper death [51]. We detected the key gene *lpdA* of cuproptosis-like death via RT-qPCR and confirmed that the *lpdA* gene level was down-regulated after Ag-CuO + H_2_O_2_ treatment, that is, the occurrence and existence of cuproptosis-like death (Fig. 8D).

To determine which component plays a role in promoting cuproptosis-like death, we set up 6 groups to detect Cu^+^ in bacterial cells. The results showed that CuO alone could cause partial Cu^+^ accumulation, while the Ag-CuO nanozymes had a better effect than CuO, indicating that the existence of Ag^+^ promoted Cu^+^ accumulation, and the amount of Cu^+^ was greater in the Ag-CuO + H_2_O_2_ group, which proved that Ag^+^ and CuO cooperated with antibacterial activity (Fig. 8F and G). Specifically, Ag^+^ can disrupt the integrity of bacterial cell membranes, thereby facilitating the rapid entry of Cu^2+^ into bacterial cells and accelerating its reduction to Cu^+^. Moreover, the catalytic activity of CuO nanozymes can be enhanced by Ag^+^, which further promotes the production of ROS and synergistically amplifies the antibacterial effect with Cu^+^-mediated cuproptosis-like death.

The above experimental results show that Ag-CuO nanozymes can not only accumulate the antibacterial activity of ROS but also down-regulate genes (*manX/Y/Z* and *fruA/B/K*) to regulate the PTS transferase system, which reduces the protection of the bacteria itself, promotes an increase in Cu^2+^ influx and Cu^+^ accumulation, and amplifies the cuproptosis-like death of bacteria (down-regulation of the *lpdA* gene). Previous studies have developed diverse antibacterial nanozymes, including Ru single-atom catalysts with NIR-II-triggered oxidase-peroxidase cascades, trimetallic Pt-PdAu alloy nanozymes, and monometallic Cu-based nanozymes that induce bacterial cuproptosis-like death [52–54]. Among them, Ru single-atom nanozymes mainly rely on photothermally enhanced ROS generation to kill bacteria, while trimetallic Pt-Pd-Au nanozymes achieve synergistic sterilization through multicomponent ROS cascade amplification. Although the latest Cu-based nanozymes have proposed the concept of cuproptosis-like death in bacteria, they only focus on single copper-mediated metabolic damage and lack the intervention of bacterial energy metabolism and synergistic effects of other metals. Compared with single-metal or single-pathway antibacterial nanozymes, Ag-CuO nanozymes integrate oxidative stress, metabolic starvation, and death-like cuproptosis into a continuous, self-enhanced amplification effect. This design not only improves the sterilization efficiency of drug-resistant bacteria but also fundamentally reduces the possibility of bacterial resistance. This new antibacterial mechanism of ROS starvation/cuproptosis could provide new ideas for the treatment of burn-infected wounds.

### Ag-CuO nanozymes promote wound healing in burn-infected mice

**Fig. 9.**
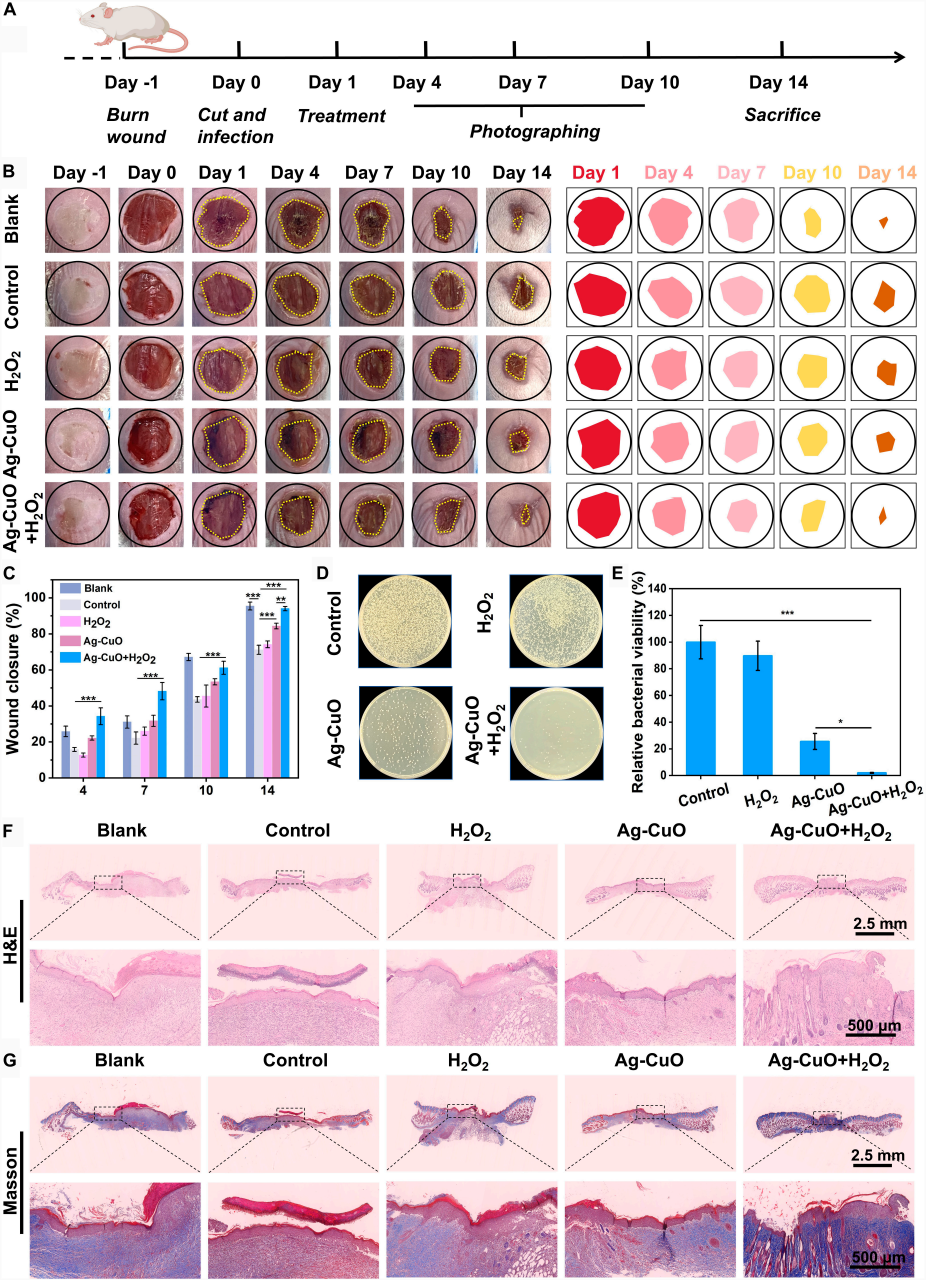
Ag-CuO nanozymes promoted wound healing in burn-infected mice. (A) Mechanistic diagram of the construction of burn wounds infected with *E. faecalis*. (B) Photos and areas of the wounds on days 1, 4, 7, 10, and 14. (C) Wound healing rates of the mice on days 4, 7, 10, and 14. (D and E) Bacterial coating and statistics of wound secretions. (F) H&E staining on day 14. (G) Masson staining on day 14. The error bars represent the means ± SDs (*n* = 3).

Burn wounds are generally divided into 3 degrees according to depth, and a 3-degree quartering method (I degree, shallow II degree, deep II degree, and III degree) is adopted. The reticular layer of the dermis in deep degree II scalding is necrotic, forming exudates rich in protein, which easily becomes bacterial culture medium [55]. To simulate deep II degree burn wounds in the clinic, we used a metal probe at 90 °C to contact the back skin of the mice for 8 s, thus establishing deep II degree burn wounds in the mice. After 24 h, the necrotic tissue was removed, and 20 μl of the standard suspension of *E. faecalis* (concentration of 1 × 10^6^ CFU/ml) was gently scraped and smeared on the scald area for 1 min until the bacterial mixture was absorbed, simulating common bacterial infection after burn. At present, many reports on MRSA and *P. aeruginosa* infection in burn wounds have been published, and the mechanism involves long-term inflammation and inability of the wound to heal. There are few studies on the mechanism by which *E. faecalis* causes burn wound healing, so we established 5 groups: the blank group (burn), the control group (burn + infection), the H_2_O_2_ treatment group (burn + infection + H_2_O_2_), the Ag-CuO treatment group (burn + infection + Ag-CuO), and the Ag-CuO + H_2_O_2_ treatment group (burn + infection + Ag-CuO + H_2_O_2_). Wound healing was evaluated by the wound healing rate, wound colony formation, hematoxylin and eosin (H&E) staining, and Masson’s trichrome staining. Inflammation- and extracellular matrix-related indicators were detected by immunofluorescence (Fig. 9A).

The results showed that by strictly controlling the intensity, time, and temperature, the scalding depth of all the wounds was deep II. According to the photos and wound size data, on the 14th day, the healing rate of the control group was only 71.1%, that of the Ag-CuO treatment group was 84.4%, and that of the Ag-CuO + H_2_O_2_ treatment group was 94.0%, which was close to that of the blank group (95.4%). Compared with the blank group, the control group healed more slowly after *E. faecalis* infection. Viscosity exudation and slight redness were observed in the wounds of the control group and H_2_O_2_ group. Ag-CuO nanozymes alone can accelerate wound healing, but the Ag-CuO + H_2_O_2_ treatment has the best effect, which may be due to the chemical kinetics effect of the Ag-CuO nanozymes combined with H_2_O_2_ (Fig. 9B and C). On the 14th day, secretions from the wound surface were coated. The results revealed that there were more bacteria in the control group and H_2_O_2_ group than in the other groups, and the Ag-CuO + H_2_O_2_ treatment had the greatest effect (Fig. 9D and E).

**Fig. 10.**
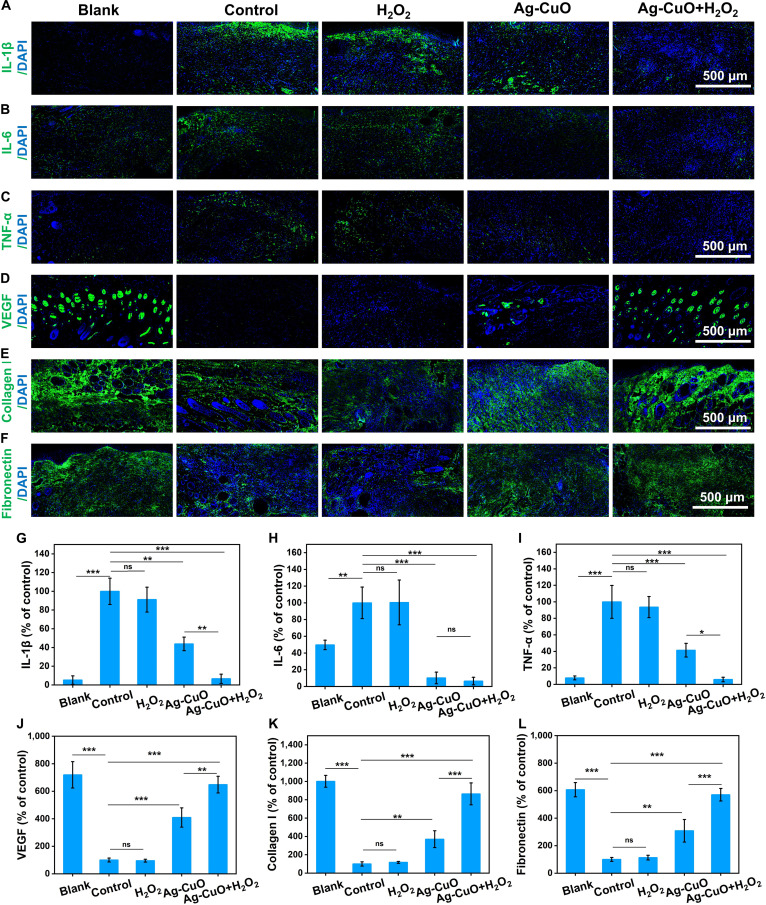
Ag-CuO nanozymes can reduce inflammation and promote angiogenesis and collagen deposition. (A and G) Immunofluorescence analysis of IL-1β and statistical analysis. (B and H) Immunofluorescence analysis of IL-6 and statistical analysis. (C and I) Immunofluorescence analysis of TNF-α and statistical analysis. (D and J) Immunofluorescence analysis of VEGF and statistical analysis. (E and K) Immunofluorescence analysis of COL I and statistical analysis. (F and L) Immunofluorescence analysis of FNs and statistical analysis. The error bars represent the means ± SDs (*n* = 3).

To further evaluate the histology of the wounds, we used H&E and Masson staining. H&E staining revealed that, compared with the blank group, the control group and the H_2_O_2_ group exhibited markedly inflammatory cell infiltration and epithelial rupture around the wound. After Ag-CuO + H_2_O_2_ treatment, the number of inflammatory cells in the wound decreased significantly, and the regeneration of skin appendages such as hair follicles and blood vessels was obvious (Fig. 9F and Fig. S6). Compared with those in the blank group, the number of collagen fibers around the wounds in the control group and H_2_O_2_ group decreased significantly, and the degree of collagen fiber deposition in the wounds increased significantly after Ag-CuO + H_2_O_2_ treatment (Fig. 9G and Fig. S7). To further verify that Ag-CuO nanozymes can reduce wound inflammation, promote angiogenesis, and remodel the extracellular matrix, we evaluated these effects via immunofluorescence staining. Compared with those in the blank group, the levels of IL-1β, IL-6, and TNF-α around the wounds in the control group and H_2_O_2_ group increased significantly, whereas the levels of inflammatory factors in the wounds decreased after Ag-CuO nanozyme treatment, and the effect in the Ag-CuO + H_2_O_2_ treatment group was the greatest (Fig. 10A to C and G to I). Compared with the blank group, the control group and H_2_O_2_ group presented less angiogenesis and increased angiogenesis after Ag-CuO nanozyme treatment, which may be related to the effect of Cu^2+^ on angiogenesis, whereas the Ag-CuO + H_2_O_2_ treatment group presented the greatest effect because inflammation was relieved (Fig. 10D and J). COL I is the most abundant collagen protein in the human body and the main mechanical supporting component of the ECM, which provides a structural framework for fibroblast migration and proliferation [56]. COL I can promote the formation of granulation tissue, and if its synthesis is reduced, it will lead to delayed healing and a fragile wound surface. Fibronectin (FN) and fibrin form a “temporary ECM” in early wounds, which guides the location of repair cells, provides a template for the deposition of COL I and COL III, and affects fiber arrangement [57]. Compared with those in the blank group, the levels of COL I and FN in the control group and H_2_O_2_ group decreased significantly, whereas the levels of COL I and FN increased significantly after Ag-CuO + H_2_O_2_ treatment, which may be related to the function of *E. faecalis* as a matrix metalloproteinase (MMP) [58] (Fig. 10E, F, K, and L).

**Fig. 11.**
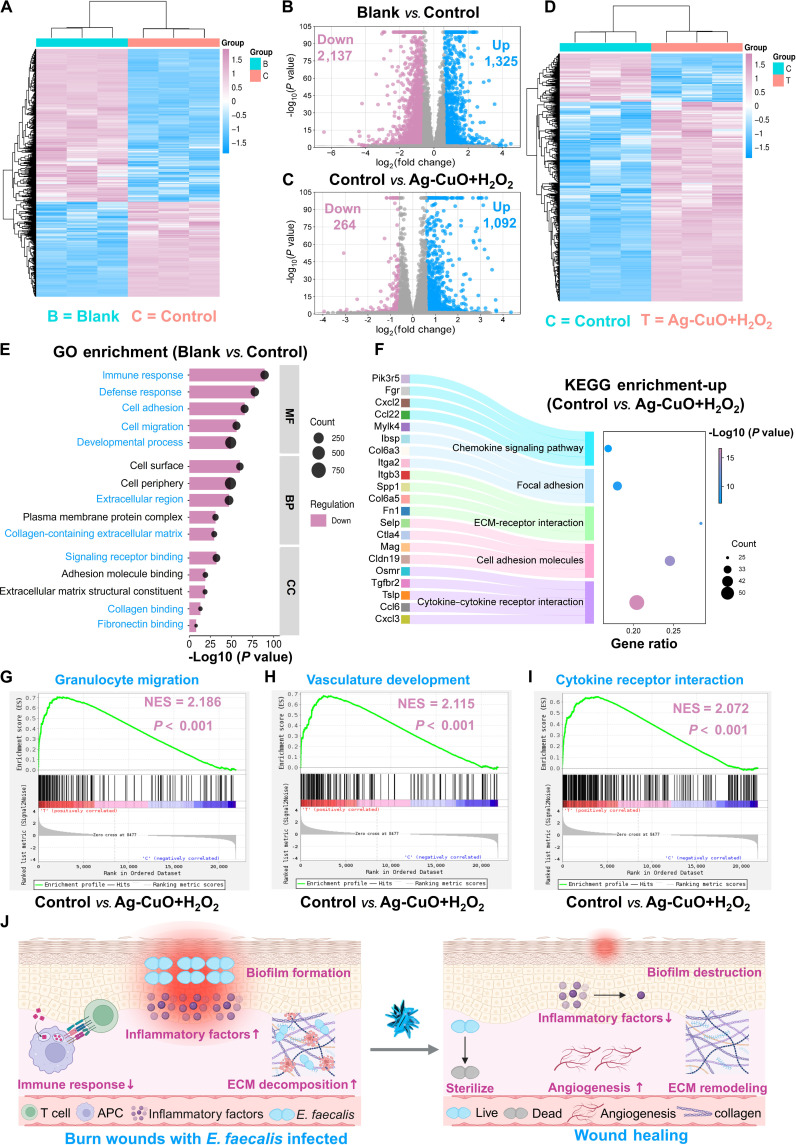
RNA sequencing of wound samples from the blank vs. control groups and the control vs. Ag-CuO + H_2_O_2_ groups (*n* = 3). (A) Heatmap of significant genes after *E. faecalis* infection. (B) Volcano maps of genes after *E. faecalis* infection. (C) Volcano maps after Ag-CuO + H_2_O_2_ treatment. (D) Heatmap after Ag-CuO + H_2_O_2_ treatment. (E) GO enrichment-All of the blank vs. control samples. (F) KEGG enrichment after Ag-CuO + H_2_O_2_ treatment. (G to I) Gene set enrichment analysis (GSEA) revealed significant differences after Ag-CuO + H_2_O_2_ treatment. (J) The mechanism by which *E. faecalis* hinders healing and Ag-CuO nanozymes promote wound healing.

To further explore the mechanism by which *E. faecalis* causes slow healing of burn wounds and by which Ag-CuO nanozymes promote wound healing, we selected fresh wound samples for transcriptome sequencing. The blank and control groups and the control and Ag-CuO + H_2_O_2_ groups were compared for comparative analysis. DEG thermography revealed that there was a significant difference in gene set enrichment between the blank group and the control group (Fig. 11A). After treatment with Ag-CuO + H_2_O_2_, the Ag-CuO + H_2_O_2_ group and the control group also presented significant differences in gene set enrichment (Fig. 11D). The results of the volcano map revealed that, compared with those of simple burn wound samples, the sequencing of wound tissue samples infected with *E. faecalis* resulted in the down-regulation of 2,137 genes and the up-regulation of 1,325 genes (Fig. 11B). After treatment with Ag-CuO + H_2_O_2_, 264 genes were down-regulated, and 1,092 genes were up-regulated (Fig. 11C). GO enrichment analysis revealed that, compared with those in the blank group, the down-regulated DEGs were enriched mainly in the immune response, defense response, and collagen-containing extracellular matrix (Fig. 11E). KEGG enrichment analysis revealed that the cytokine–cytokine receptor interaction and ECM–receptor interaction signaling pathways were down-regulated in the control group compared with those in the blank group (Fig. S5). Compared with those in the control group, the genes whose expression was up-regulated in the Ag-CuO + H_2_O_2_ group were enriched mainly in cell adhesion molecules, the extracellular matrix, and the cytokine signaling pathway (Fig. 11F). GSEA revealed that after treatment with Ag-CuO + H_2_O_2_, the granulocyte migration, angiogenesis, and cytokine interaction pathways were up-regulated (Fig. 11, G to I), which was consistent with the immunofluorescence results.

On the basis of the above experimental results, we preliminarily determined that once a burn wound is infected with *E. faecalis*, *E. faecalis* acts as an MMP after it forms a biofilm on the wound, which overwhelms the collagen and fibronectin needed for wound healing, making healing difficult. In addition, the immune escape of *E. faecalis* makes the body unable to effectively remove bacteria, resulting in an inflammatory state for a long period of time [59]. After Ag-CuO nanozyme treatment, it can effectively clear the bacterial biofilm of infected wounds, restore inflammatory homeostasis and eliminate the state of overdecomposition of proteins, thus remodeling the ECM [60]. In addition, the Ag-CuO nanozymes effectively promoted vascular growth and accelerated wound healing (Fig. 11J).

### Ag-CuO nanozymes promote wound healing in burn-infected New Zealand rabbits

**Fig. 12.**
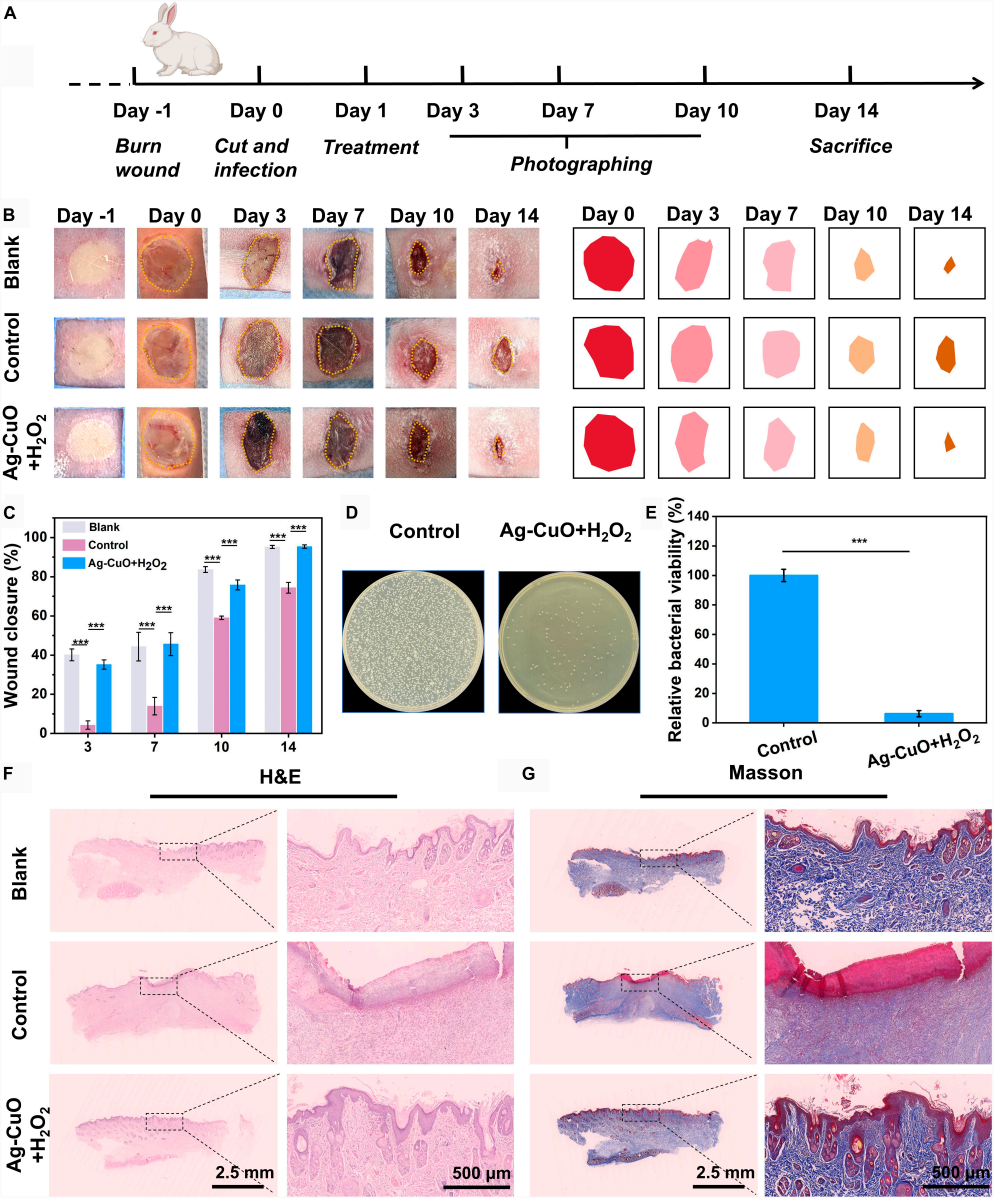
Ag-CuO nanozymes promoted wound healing in burn-infected New Zealand rabbits. (A) Mechanistic diagram of the construction of burn wounds infected with *E. faecalis*. (B) Photos and areas of the wounds on days 0, 3, 7, 10, and 14. (C) Wound healing rates of rabbits on days 3, 7, 10, and 14. (D and E) Bacterial coating and statistics of wound secretions. (F) Hematoxylin and eosin (H&E) staining on day 14. (G) Masson staining on day 14. The error bars represent the means ± SDs (*n* = 3).

Owing to ethical limitations and the particularity of burn wounds, it is difficult to carry out human experiments to understand the antibacterial activity of Ag-CuO nanozymes in the human body and their ability to promote burn wound healing. We conducted experiments in mice to preliminarily verify its effectiveness. However, the mouse epidermis is thinner, the human epidermis is thicker (multiple layers, especially the stratum corneum, are more developed), and the density of collagen and accessory structures (such as hair follicles and sebaceous glands) in the mouse dermis is different from that in humans, which may affect the speed of wound healing. The limitations of these studies cannot be ignored. The thickness of the epidermis and dermis (especially the back skin) of New Zealand rabbit skin is between that of mouse skin and human skin, which is closer to that of some parts of human skin (such as limbs) and is suitable for simulating II degree scald skin. The healing cycle (2 to 3 weeks) of some deep scalds (such as those with a depth of II degrees) is similar to that of humans. Therefore, we established a New Zealand rabbit scald infection model to further verify the antibacterial activity of Ag-CuO nanozymes in vivo and their ability to promote burn wound healing (Fig. 12A).

The results revealed that the scalding depth of all the wounds was deep II degrees. According to the photos and wound size data, on the 14th day, the wound healing rate of the control group was only 74.3%, and that of the Ag-CuO + H_2_O_2_ treatment group was 95.4%, which was close to that of the blank group (95.2%). Unlike the wounds of the control group of New Zealand rabbits, the wounds of the control group of New Zealand rabbits presented obvious redness and exudation, which is very similar to the state of human skin infection. In the Ag-CuO + H_2_O_2_ treatment group, there was no obvious redness or swelling around the wound, and the wound was clean and free of exudate (Fig. 12B and C). On the 14th day, the secretions were removed from the wound surface and coated. The results revealed that there were more bacteria in the control group, accompanied by miscellaneous bacteria, which was consistent with the clinical mixed infection of the wound surface. The Ag-CuO + H_2_O_2_ treatment effectively eliminated bacteria, but some bacteria were still present (Fig. 12D and E). The results of H&E staining revealed that, compared with those in the blank group, there was obvious inflammatory cell infiltration around the wounds in the control group, but no obvious inflammatory cells were found in the wounds after Ag-CuO + H_2_O_2_ treatment, accompanied by angiogenesis (Fig. 12F and Fig. S8). Masson staining revealed that, compared with the control, Ag-CuO + H_2_O_2_ effectively promoted collagen deposition (Fig. 12G and Fig. S9). These results are consistent with those in mice. This series of progressive animal experiments (mice-New Zealand rabbits) will strengthen the verification results; that is, Ag-CuO nanozymes can not only eliminate bacteria and reduce inflammation but also promote angiogenesis and collagen deposition and accelerate wound healing.

### Biosafety assessment of Ag-CuO nanozymes

Before the in vivo experiments, we tested the biosafety of these compounds using cell counting kit-8 (CCK8) and a hemolysis assay in human umbilical vein endothelial cells (HUVECs). CCK8 experiments revealed that when the concentration of Ag-CuO nanozymes was 10 μg/ml, the cell activity was 91.6% of that of the control group; when the concentration of Ag-CuO nanozymes was 40 μg/ml, the cell activity was 84.2% of that of the control group (Figs. S10 and S11). The results of the hemolysis experiments revealed that when the concentration of Ag-CuO nanozymes was less than 30 μg/ml, the hemolysis rate was less than 5%. According to in vitro probe efficacy detection and antibacterial tests, our selected concentration (8 μg/ml) is within the safe range. On the 14th day of the experiment in vivo, we performed H&E staining on the heart, liver, spleen, lung, and kidney of all groups of mice, and the results revealed no obvious lesions (Fig. S12). The routine blood and biochemical indices of the mice were all within the normal range (Figs. S13 and S14). The above results indicated that 8 μg/ml Ag-CuO nanozymes had good biosafety in vivo. The negligible in vivo toxicity can be attributed to the rational dosage selection within the safety threshold, the pH-dependent catalytic behavior that confines ROS generation mainly to acidic infectious microenvironments rather than healthy tissues, and the good biocompatibility and nonaccumulative nature of the Ag-CuO nanozymes in major organs.

### Therapeutic effect of the Ag-CuO nanozyme versus conventional clinical treatment

The efficacy of Ag-CuO nanozymes in the treatment of infected wounds after burn injury has been verified by the above experimental results. To further explore the feasibility of its clinical application, we comprehensively compared its efficacy with that of clinical medication through in vivo and in vitro experiments. This research has focused mainly on the biological safety, minimum inhibitory concentration (MIC), drug resistance, and treatment effect of drug-resistant bacterial infection and other aspects of comprehensive evaluation.

Resazurin staining for the determination of the MIC is a widely used and very classical microbiological method. The core of this principle is to detect the metabolic activity of bacteria by using resazurin as a redox indicator. We incubated 100 μl of the negative control group (phosphate-buffered saline [PBS]), positive control group (vancomycin [Van], 1 mg/ml), or drug control group (equal gradient concentration) with 100 μl of the *E. faecalis* standard strain (optical density [OD] = 0.1) for 18 h and then incubated the mixture with resazurin staining solution for 2 h. The results revealed that the MICs of the Ag-CuO nanozymes, Van, and the hydrofiber dressing with silver (Hydrofiber@Ag) were 8, 2, and 5 μg/ml, respectively (Fig. S15). Among the 3 drugs, Van has the lowest dosage and the least toxicity to cells. Even when the concentration of Van was 256 μg/ml, the toxicity and side effects on cells were not obvious, which may be related to the bactericidal mechanism by which Van inhibits the synthesis of the bacterial cell wall (Figs. S16 and S17).

**Fig. 13.**
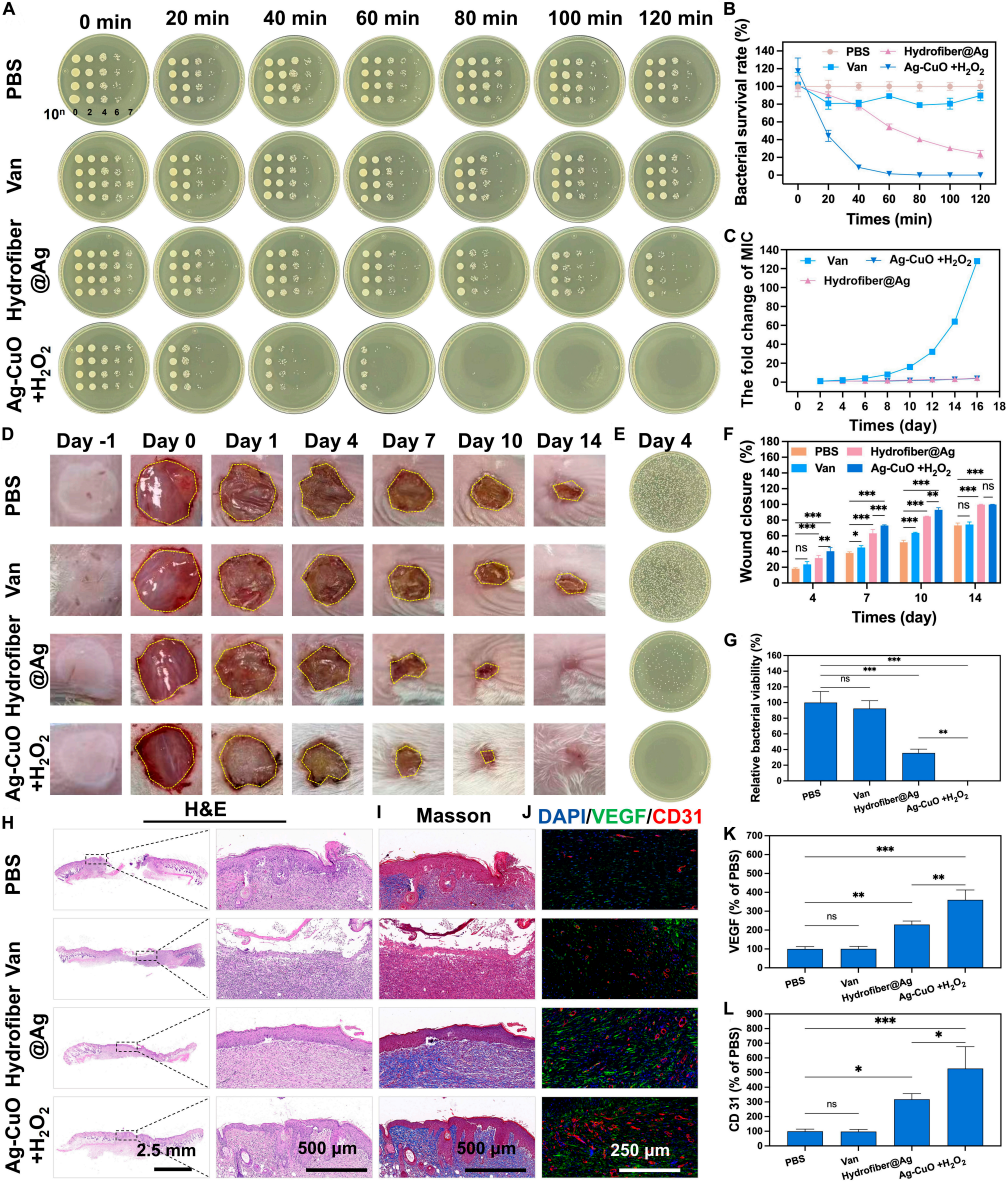
Evaluation of the efficacy of Ag-CuO nanozymes and clinical drugs in killing VRE. (A and B) Killing effects and curves of different groups of drugs on VRE within 120 min (*n* = 4). (C) Fold change in the drug resistance concentration of VRE in response to different drugs (*n* = 4). (D and F) Photos and wound healing rates of the wounds on days −1, 0, 1, 4, 7, 10, and 14 (*n* = 3). (E and G) Bacterial coating and statistics of wound secretions. (H and I) H&E and Masson staining on day 14 (*n* = 3). (J to L) Immunofluorescence images and statistical analysis of VEGF and CD31 expression (*n* = 3). The error bars represent the means ± SDs.

To detect the ability of drugs to kill drug-resistant bacteria, we first cultured VRE (256 μg/ml) through 16 days of rapid screening and then established an antibacterial experimental model to verify its efficacy. The results of the inhibition zone revealed that Van at a mass of 32 μg had no obvious inhibitory effect on VRE, which met the international general standards and verified the successful establishment of VRE strains. The Ag-CuO + H_2_O_2_ mixture at equal concentrations had excellent inhibitory effects on VRE. When the mass of Ag-CuO was 32 μg, the average antibacterial diameter of VRE was 1.9 cm, and VRE was sensitive to the Ag-CuO + H_2_O_2_ group. To compare the effects of Ag-CuO and Hydrofiber@Ag more accurately, we controlled the silver ion content to compare the bacteriostatic ring effect (the mass ratio of Ag in Hydrofiber@Ag was 1.2%). The results showed that the Ag-CuO + H_2_O_2_ group had better antibacterial effects than the Hydrofiber@Ag group at the same silver ion content (Fig. S18). To more intuitively reflect the killing effect of the 3 drugs on VRE, we continuously sampled the incubated bacterial solution within 120 min and then coated the plate to observe bacterial growth. The results showed that Van had no obvious killing effect on VRE. The Ag-CuO + H_2_O_2_ group and the Hydrofiber@Ag group with the same content of silver ions showed excellent killing effects. Among them, the Ag-CuO + H_2_O_2_ group had the best effect and almost completely killed the VRE at 60 min (Fig. 13A and B). In addition, to further compare the resistance of standard strains of *E. faecalis* caused by the 3 drugs, we tested the MIC by screening Van-resistant *E. faecalis* strains at equal concentrations. Even when the resistance concentration of *E. faecalis* to Van reached 256 μg/ml, Ag-CuO and Hydrofiber@Ag still killed *E. faecalis*, and the fold change in the MIC was within 5 (Fig. 13C). The unique bactericidal mechanism of Ag-CuO and hydrofibers@Ag makes bacterial resistance difficult.

To compare the efficacy of the 3 drugs in vivo, we selected BALB/c mice to establish a burn wound model with VRE infection. The results showed that the Van group had no obvious effect on promoting wound healing in vivo, and there was no significant difference in wound healing time between the Van group and the PBS group. Compared with the PBS and Van groups, the Ag-CuO + H_2_O_2_ and Hydrofiber@Ag groups presented excellent wound healing properties. At the same silver ion content, the plants in the Ag-CuO + H_2_O_2_ group healed faster than those in the Hydrofiber@Ag group on days 4, 7 and 10, but both groups completed healing on day 14. This may be due to the role of sodium carboxymethyl cellulose in Hydrofiber@Ag, which quickly completes epithelization during the remodeling phase (Fig. 13D and F). On the fourth day, the antibacterial effect of the Ag-CuO + H_2_O_2_ group was greater than that of the Hydrofiber@Ag group (Fig. 13E and G). The results of HE and Masson staining revealed that the PBS group and the Van group contained many inflammatory cells, the granulation tissue was missing, and the degree of collagen synthesis and deposition was insufficient. Complete epithelial regeneration was observed in the Ag-CuO + H_2_O_2_ and Hydrofiber@Ag groups, without continuous or massive inflammatory cell infiltration. Extensive, bright blue-stained areas were observed in the wound area, indicating that a large amount of collagen was synthesized and deposited (Fig. 13H and I and Figs. S19 and S20). In particular, the antiangiogenic effect of Ag-CuO + H_2_O_2_ was greater than that of the Hydrofiber@Ag group, which may be related to the role of copper ions in Ag-CuO (Fig. 13J to L). In addition, the organs of the mice treated with the drug for 2 weeks were examined, and the results revealed that the 3 drugs had no obvious toxicity or side effects in the short term (Fig. S21).

The above experimental results show that although Van has high biosafety and is effective for standard strains, it easily induces drug resistance, resulting in its ineffectiveness for VRE, and systemic medication has difficulty reaching an effective concentration in wounds, so it is not suitable for local wound treatment. With the same silver content, the bactericidal speed and efficacy of the Ag-CuO nanozyme are better than those of Hydrofiber@Ag, and it is not easy to resist and has the unique advantage of promoting angiogenesis. However, it is difficult for simple nanoparticles to effectively remain in wounds. Although the direct bactericidal ability of Hydrofiber@Ag in clinical application is slightly worse than that of Ag-CuO, its carboxymethylcellulose sodium matrix can provide an ideal moist environment for wound epithelization, and the comprehensive healing effect is excellent. Therefore, if Ag-CuO nanozymes are combined with advanced dressing matrices such as hydrofibers, the development of next-generation wound dressings with strong antibacterial, nonresistant, and active healing functions is expected [61–63]. Such composite dressings can not only effectively address the clinical challenges of drug-resistant bacterial infections in wounds but also avoid the side effects caused by excessive use of antibiotics or traditional silver-based dressings. Moreover, the unique dual-metal synergistic effect of Ag-CuO nanozymes can maintain long-term antibacterial activity while promoting wound tissue regeneration, making them highly suitable for the treatment of various difficult-to-heal wounds, such as burn infections and diabetic ulcers.

## Conclusion

In this study, a uniform and flower-like bimetallic nanozyme (Ag-CuO) was successfully synthesized. Ag-CuO nanozymes possess POD-like, OXD-like, and GSH-Px-like enzyme activities, which can produce ROS and reduce ROS clearance, thus destroying the bacterial cell membrane and causing LPO damage. In addition, Ag-CuO nanozymes down-regulate the levels of the *manX/Y/Z* and *fruA/B/K* genes to regulate PTS, reduce the carbohydrate intake required for bacterial energy synthesis, and down-regulate the TCA cycle to reduce ATP synthesis, thereby inhibiting its own protection against external stimuli. Finally, owing to the down-regulation of the *lpdA* gene by Ag-CuO nanozymes and the inability of bacteria to resist external stimuli, Cu^2+^ influx and Cu^+^ accumulation increased, thus amplifying the cuproptosis-like death and biofilm inhibition of bacteria. The unique ROS-starvation/cuproptosis-like death sterilization method overcomes the limitation that traditional antibiotics are easily tolerated by bacteria. Importantly, the efficacy gradually verified in a mouse New Zealand rabbit model strongly demonstrated its potential for clinical use in drug-resistant bacterial infections, as it has excellent antibacterial activity and the ability to promote angiogenesis compared with those of commercial silver-based dressings with the same silver ion content. The bimetallic nanozyme (Ag-CuO) introduced in this study can resist bacteria via multiple mechanisms, which provides a new solution for the treatment of drug-resistant bacteria after burn injury.

## Materials and Methods

### Synthesis of Ag-CuO nanozymes

The synthesis was initiated by dissolving CuCl₂·H₂O (1.3400 g) and PEG (1.0000 g) in 30 ml of distilled water, followed by the addition of 2 ml of hydrogen peroxide under vigorous stirring for 20 min. A 30-ml aliquot of 2 M NaOH solution was then introduced dropwise over a 30-min period. After the reaction mixture was heated to 90 °C for 30 min, 50 ml of AgNO₃ solution (0.1 g in water) was added dropwise with continuous stirring. The resulting precipitate was isolated by centrifugation at 18,000 rpm for 30 min, subjected to 3 washing cycles with deionized water and ethanol under ultrasonication, and finally vacuum-dried at 60 °C for 12 h [33].

### Characterization of the Ag-CuO nanozymes

The morphology and particle size of the Ag-CuO nanozymes were observed via SEM (ZEISS Gemini SEM 300, Germany), TEM (Japan-JEOL-JEM 2100 F), and HRTEM. The lattice price was observed via XRD (Bruker, D8 Advanced Diffractometer) and selected area electron diffraction. The valence state and elemental analysis were carried out via XPS (USA—Thermo Fisher + ESCALAB Qxi). The elemental content was quantified via ICP (EXPEC 6500).

### Detection of multienzyme activity

The POD-like and OXD-like activities of Ag-CuO nanozymes were detected with TMB and OPD probes. The concentrations of H_2_O_2_ (0, 0.2, 0.4, 0.6, 0.8, and 1.0 mM), Ag-CuO nanozymes (0, 2, 4, 6, 8, and 10 μg/ml), and different groups were determined. The pH of all the solutions was 5.5. The activity of GSH-Px-like was detected by a DTNB probe, and the consumption rates of the Ag-CuO nanozymes at 4 μg/ml were detected at different time points (0, 10, 30, 60, 120, 180, 240, and 300 min). When the concentration of the Ag-CuO nanozymes was 10 μg/ml, the consumption rates of the Ag-CuO nanozymes during different time periods (0, 5, 10, 30, and 60 min) were measured. The pH of all the solutions was 5.5, *n* = 3.

### In vitro antibacterial performance test

#### Plate counting method

First, all operation specifications must be ensured to avoid cross-contamination. After the purchased bacterial powder was activated, it was marked as a seed plate, and 3 to 4 single colonies were incubated overnight in broth, ensuring an OD_600_ = 0.6 as much as possible. When hydrogen peroxide (100 μM) was added or not added, different concentrations of 0, 2, 4, 6, and 8 μg/ml Ag-CuO nanozymes and bacteria were incubated in a PBS solution at pH 5.5. After 4 h, the bacterial mixture was diluted 1,000 times, and 40 μl of diluted bacterial mixture was added onto an agar plate. After incubation overnight, the samples were photographed, and the survival rate was calculated via ImageJ software. Two gram-positive bacteria (MRSA and *E. faecalis*) and 2 gram-negative bacteria (*P. aeruginosa* and *E. coli*) were used in the experiment (*n* = 3).

#### Bacterial growth curves

Different groups (control, H_2_O_2_, AgNO_3_, CuO, Ag-CuO, and Ag-CuO + H_2_O_2_) were established, the concentration of the Ag-CuO nanozymes was 8 μg/ml, and the molar concentrations of AgNO_3_ and CuO were consistent with those of the Ag-CuO nanozymes. The OD_600_ of the initial bacterial mixture was 0.1, and the blank controls of broth, PBS, AgNO_3_, CuO, Ag-CuO, and Ag-CuO + H_2_O_2_ were set accordingly. The OD values were measured at 0, 2, 4, 6, 8, 10, 12, and 14 h via a microplate reader (SpectraMax iD3), and the growth curves were drawn via Origin. Two gram-positive bacteria (MRSA and *E. faecalis*) and 2 gram-negative bacteria (*P. aeruginosa* and *E. coli*) were used in the experiment (*n* = 3).

#### Bacterial live/dead staining

For bacterial live/dead staining, 4 groups (control, H_2_O_2_, Ag-CuO, and Ag-CuO + H_2_O_2_) were set up, the concentration of Ag-CuO nanozymes was 8 μg/ml, the initial bacterial solution OD_600_ was 0.6, and after incubation for 4 h, N01/PI staining was used for 25 min. Photos were then taken under a confocal laser scanning microscope (CLSM, CSIM-130), and the data were processed with ImageJ software. The ROS staining reagent used was DCFH-DA, the LPO staining reagent used was the BODIPY 581/591 C11 fluorescence staining kit, and the bacterial treatment used was the same as that used for live/dead staining, *n* = 3.

#### Inhibition of bacterial biofilms

Four groups (control, H_2_O_2_, Ag-CuO, and Ag-CuO + H_2_O_2_) were set up, the concentration of the Ag-CuO nanozymes was 8 μg/ml, and the initial bacterial solution OD_600_ was 0.6. All samples were incubated in 24-well plates for 48 h and observed via crystal violet staining, live/dead staining, and SEM (*n* = 3).

#### Destruction of the bacterial biofilm

For the destruction of the bacterial biofilm, the initial bacterial mixture (OD_600_ = 0.6) was first incubated in a 24-well plate for 48 h to form a biofilm, after which 4 groups (control, H_2_O_2_, Ag-CuO, and Ag-CuO + H_2_O_2_) were established. Ag-CuO nanozymes at a concentration of 8 μg/ml were incubated for 24 h, and the biofilms were observed via crystal violet staining, live/dead staining, and SEM (*n* = 3).

#### Cu^+^ fluorescence staining

Six groups (control, H_2_O_2_, AgNO_3_, CuO, Ag-CuO, and Ag-CuO + H_2_O_2_) were established. The concentration of the Ag-CuO nanozymes was 8 μg/ml, and the molar concentrations of AgNO_3_ and CuO were the same as those of the Ag-CuO nanozymes. Initial bacterial solution OD_600_ = 0.6. After incubation for 4 h, Coppersensor 1 was used for staining for 5 min, and then photographs were taken via CLSM. ImageJ was used to process the data, *n* = 3.

### Evaluation of antibacterial and wound healing properties in vivo

#### Model of burn-induced *E. faecalis* infection in mice

Thirty BALB/c mice aged 6–8 weeks were randomly divided into 5 groups (6 mice in each group): the blank group (burn), the control group (burn + infection group), the H_2_O_2_ treatment group (burn + infection + H_2_O_2_ group), the Ag-CuO treatment group (burn + infection + Ag-CuO group), and the Ag-CuO + H_2_O_2_ treatment group (burn + infection + Ag-CuO + H_2_O_2_ group). The bacterial distribution and wound healing of the wounds were observed.

#### Model of *E. faecalis* infection in New Zealand rabbits

Six New Zealand rabbits were used, each of which had 3 wounds on the back, 1 on the left back, and 2 on the right back. To avoid bacterial contamination, the wounds on the left back were set as the blank group, and the 2 wounds on the right were randomly divided into a control group (burn + infection group) and a treatment group (burn + infection + Ag-CuO + H_2_O_2_ group), which were covered with gauze before and after treatment. The wound bacterial distribution and wound healing were observed.

#### Biosafety of Ag-CuO nanozymes

To determine the biocompatibility of Ag-CuO nanozymes, CCK8 and hemolysis tests were used to detect cell safety, and biosafety was evaluated according to heart, liver, spleen, lung, and kidney tissue sections and the hematological indices of the animals.

### Evaluation of the efficacy of Ag-CuO nanozymes and clinical drugs

#### The MIC was determined via the resazurin method

We incubated 100 μl of the negative control group (PBS), positive control group (Van, 1 mg/ml), or drug control group (equal gradient concentration) with 100 μl of the *E. faecalis* standard strain (OD = 0.1) for 18 h and then incubated the mixture with resazurin staining solution for 2 h.

#### Cultivation of drug-resistant bacteria

When the MIC of Van for *E. faecalis* was 2 μg/ml, an equal volume of Van was added on the first day, and the bacteria were cultured with the bacterial mixture for 18 h until the concentration of Van was 2 μg/ml. On the second day, the bacteria were streaked, and the bacteria growing on the plate were *E. faecalis* resistant to 2 μg/ml. On the third day, the concentration of Van was increased to 4 μg/ml by adding an equal volume of Van to the bacterial mixture for 18 h. On the 4th day, the bacteria were streaked, and the bacteria growing on the plate were *E. faecalis* resistant to 4 μg/ml. Similarly, 256 μg/ml VRE was obtained on the 16th day.

#### Model of burn-induced VRE infection in mice

Twenty-four BALB/c mice aged 6 to 8 weeks were randomly divided into 4 groups (6 mice in each group): the PBS group, the Van group, the Hydrofiber@Ag group, and the Ag-CuO + H_2_O_2_ treatment group. The bacterial distribution and wound healing of the wounds were observed.

### Statistical analysis

The data are presented as the means ± SDs from a minimum of 3 independent experimental replicates. For comparisons between 2 groups, Student *t* test was employed, whereas 1-way or 2-way analysis of variance with Tukey’s post hoc test was used for multiple group comparisons. Significance thresholds were set at *P < 0.05, **P < 0.01, and ***P < 0.001.

## Ethical Approval

The animal-related procedures were carried out in conformity with the guidelines established by the Institutional Animal Care and Use Committee of Anhui Medical University (approval no. LLSC20252093). All mice were housed and euthanized in compliance with the animal research policies of the National Ministry of Health as per standard regulations.

## Data Availability

The datasets underpinning the results of this research can be obtained from the corresponding authors upon a justified request.
