# Supplementary material for: Ag-CuO Nanozymes Superior to Commercial Silver-Based Dressings: Synergistic Antibacterial Therapy via PTS-Mediated Starvation and Cuproptosis-Like Death
Source: Research (Wash D C). 2026 Jun 11;9:1297. doi: 10.34133/research.1297 (PMC13254178; doi:10.34133/research.1297)
Supplement: Supplementary 1 — Figs. S1 to S21 Table S1 [file research.1297.f1.docx]

**Supporting Information for**

**Original article**

**Ag-CuO Nanozymes Superior to Commercial Silver-Based Dressings: Synergistic Antibacterial Therapy via PTS-Mediated Starvation and Cuproptosis-Like Death**

*Shuo Zhang^1#^, Dongliang Yang^2#^, Yunfeng Zheng^1^, Yangyang Liu^3^, Xinyu Zhao^1^, Yutong He^4, 5^, Xu-Lin Chen^1^*, Xianwen Wang^4, 5^**

1. Department of Burns, The First Affiliated Hospital of Anhui Medical University, Hefei 230032, P. R. China.
2. School of Physical and Mathematical Sciences, Nanjing Tech University, Nanjing 211816, P. R. China
3. Department of Orthopedics, First Affiliated Hospital of Bengbu Medical University, Bengbu 233000, P. R. China.
4. Anhui Province Key Laboratory of Occupational Health, Anhui No. 2 Provincial People's Hospital, Hefei 230041, P. R. China.
5. School of Biomedical Engineering, Anhui Medical University, Hefei 230032, P. R. China.

***Corresponding Authors E-mail:** [xianwenwang@ahmu.edu.cn](mailto:xianwenwang@ahmu.edu.cn) (X. Wang); E-mail: [chenxulin@ahmu.edu.cn](mailto:chenxulin@ahmu.edu.cncom) (X. Chen)

**^#^**These authors contributed equally to this work.

**Experimental section**

***Transcriptomic sequencing***

The bacteria were divided into two groups (control and Ag-CuO + H_2_O_2_), and the concentration of the Ag-CuO nanozymes was 8 μg/mL. The initial bacterial mixture had an OD_600_ = 0.6, was incubated for 4 hours and then frozen for inspection (n=3). All samples were prepared according to the instructions of Personal Technology Co., Ltd. (Shanghai, China). In addition, data processing, including correlation analysis, differentially expressed gene (DEG) analysis, gene ontology (GO) analysis, and Kyoto Encyclopedia of Genes and Genomes (KEGG) analysis, was performed on a personal technology cloud platform (<https://genescloud.oss-cn-shanghai.aliyuncs.com>).

***Quantitative real-time PCR (RT‒qPCR)***

The bacteria were treated as described above, and all the samples were prepared according to the instructions of Personal Technology Co., Ltd. (Shanghai, China). The forward and reverse sequences of the primers used are listed below.

**Table S1.** qPCR primers.

| gene-WMS_RS04395 | Forward | 5' ATTCCTGCTGACACTGTT 3' |
| --- | --- | --- |
|  | Reverse | 5' CCATACCACCACCGATAG 3' |
| gene-WMS_RS04400 | Forward | 5' AGCACCACTACTACTTACAT 3' |
|  | Reverse | 5' CTACGCCGACTACGAATA 3' |
| gene-WMS_RS04390 | Forward | 5' GCGAACTCATTGTTAGAAG 3' |
|  | Reverse | 5' CTTGTGCTGATTCCATAGA 3' |
| gene-WMS_RS06445 | Forward | 5' TTGTTACTCACGCTGTTC 3' |
|  | Reverse | 5' TTGATAACGATTGCTCTTGT 3' |
| gene-WMS_RS06450 | Forward | 5' GAGAACAAGAAGGAACAA 3' |
|  | Reverse | 5' AAGGTAACGATGATAATGG 3' |
| gene-WMS_RS06630 | Forward | 5' GTCTGCTGATACACGAAT 3' |
|  | Reverse | 5' CCTGAACACGACTAACTG 3' |
| gene-WMS_RS10340 | Forward | 5' ATCGGTGCTGAATTAGGT 3' |
|  | Reverse | 5' TCTTGAAGTCGTCTGTGA 3' |
| 16S | Forward | 5' CCGAGTGCTTGCACTCAATTGG 3' |
|  | Reverse | 5' CTCTTATGCCATGCGGCATAAAC 3' |

**Evaluation of antibacterial and wound healing properties *in vivo***

***Model of Burn-induced E. faecalis infection in mice***

All animal experiments were approved by the Ethics Committee of Anhui Medical University (no. LLSC20252093). General anesthesia was induced in the mice via mixed oxygen inhalation with 1.5% isoflurane. The successful indications of anesthesia were loss of the eyelid reflex, muscle relaxation, and decreased movement. After anesthesia, a small animal depilation machine and depilation cream were used to prepare the skin. Through a customized burn instrument, a metal probe with a temperature of 90 °C was used to contact the skin of the back of each mouse, and a round burn wound with a diameter of approximately 8 mm was established on the back of each mouse for 8 seconds. After 24 hours, anesthesia was performed again, and the burn wound was cut on the back with surgical scissors to form a burn wound with a diameter of approximately 8 mm. A 20 μL standard suspension of *Enterococcus faecalis* (concentration of 1×10^6^ CFU/mL) was gently scraped and smeared on the scald site for 1 min until the bacterial mixture was absorbed, simulating common bacterial infection after burn injury. After inoculation, the wound surface was covered with sterile gauze to prevent cross infection. The next day, the corresponding treatment was carried out according to the group name. The drug concentration of Ag-CuO was 8 μg/mL (20 μL), and the H_2_O_2_ concentration was 100 μmol. On the 14th day, wound secretions were removed to create a bacterial coating plate. Histopathological H&E and Masson staining and immunofluorescence detection (*VEGF, IL-1β, IL-6, TNF-α, COL Ⅰ and FN*) were performed on the skin samples. To evaluate the degree of wound healing, the blood of each group of animals was collected for routine blood and blood biochemical analyses, and organs (heart, liver, spleen, lung and kidney) were collected for the evaluation of material biocompatibility.

***Model of burn infection with E. faecalis in New Zealand rabbits***

General anesthesia was induced in rabbits via 2% isoflurane via mixed oxygen inhalation. The successful indications of anesthesia were loss of the eyelid reflex, muscle relaxation, and decreased movement. After anesthesia, the skin was prepared with a small animal depilation machine and depilation cream. Through a customized burn instrument, the back skin of New Zealand white rabbits was contacted with a metal probe at a temperature of 90 °C, and three round burn wounds with a diameter of approximately 1 cm were established on the back of each New Zealand white rabbit for 10 seconds. After 24 hours, anesthesia was performed again, and the burn wound was cut on the back with surgical scissors to form a burn wound with a diameter of approximately 1 cm. A 40 μL standard suspension of *Enterococcus faecalis* (concentration of 1×10^6^ CFU/mL) was gently scraped and smeared on the scald site for 1 min until the bacterial mixture was absorbed, simulating common bacterial infection after burn injury. After inoculation, the wound surface was covered with sterile gauze to prevent cross infection. The next day, the corresponding treatment was carried out according to the group. The drug concentration of Ag-CuO was 8 μg/mL (40 μL), and the H_2_O_2_ concentration was 100 μmol. On the 14th day, wound secretions were removed to create a bacterial coating plate. At the same time, skin samples were taken for histopathological H&E and Masson staining.

***H&E, Masson***

After 14 days of treatment, the mice were euthanized. Cold saline and appropriate laboratory equipment were sterilized and disinfected, and tissues, including skin, muscle and other tissues associated with the wound, were collected along the periphery of the wound. The collected tissues were fixed, embedded, sectioned and stained with H&E and Masson’s trichrome. The histological features of the tissues were assessed under a light microscope, and stained images were recorded.

***Immunohistochemical staining***

The sections were incubated in 3% H_2_O_2_ at room temperature to increase endogenous peroxidase activity and washed three times each with distilled water and PBS. Primary antibodies (against *VEGF, IL-1β, IL-6, TNF-α, COL Ⅰ and FN*) were added dropwise to the sections, which were subsequently incubated at 4 °C overnight. After the samples were washed 3 times with PBS, secondary antibody was added dropwise at room temperature and incubated for 30 min. After they were washed 3 times with PBS, DAB chromogen was added for 5–10 min, and hematoxylin violet lining staining was used for 2–5 min. Differentiation with 1% hydrochloric acid alcohol was performed for 10 s, and lithium carbonate solution bluing was performed for 30 s. After routine dehydration, xylene transparency was used, the slices were sealed, and the results of the staining were observed and recorded under a microscope.

**Biosafety *in vivo***

***Histological sections***

The mice were sacrificed by cervical dislocation and dissected to remove the heart, liver, spleen, lung and kidney. The tissues were fixed with 4% paraformaldehyde, and after 24 hours, the fixed tissues were immersed in melted paraffin wax. The wax blocks were fixed in a slicer and cut into 5-μm-thick sections. H&E staining was performed on the sections, and the stained images were observed and recorded via light microscopy.

***Hematological indices***

Blood was collected from the mice via the posterior orbital venous plexus. After the mice were anesthetized, capillary blood vessels were placed in the medial corner of the eye and punctured at 30–45 degrees, which is not too deep, and blood samples were collected via anticoagulation tubes and ordinary 1.5 mL EP tubes. The blood in the anticoagulation tubes was shaken in an ‘8’ motion so that the blood was in full contact with the anticoagulant on the wall of the tubes, and a veterinary hematology analyzer was used for routine blood tests (BC-5000VET). The EP tubes were centrifuged (4000 rpm, 5 min) at 4 °C in a low-temperature centrifuge, and the supernatant was aspirated for blood biochemistry testing.


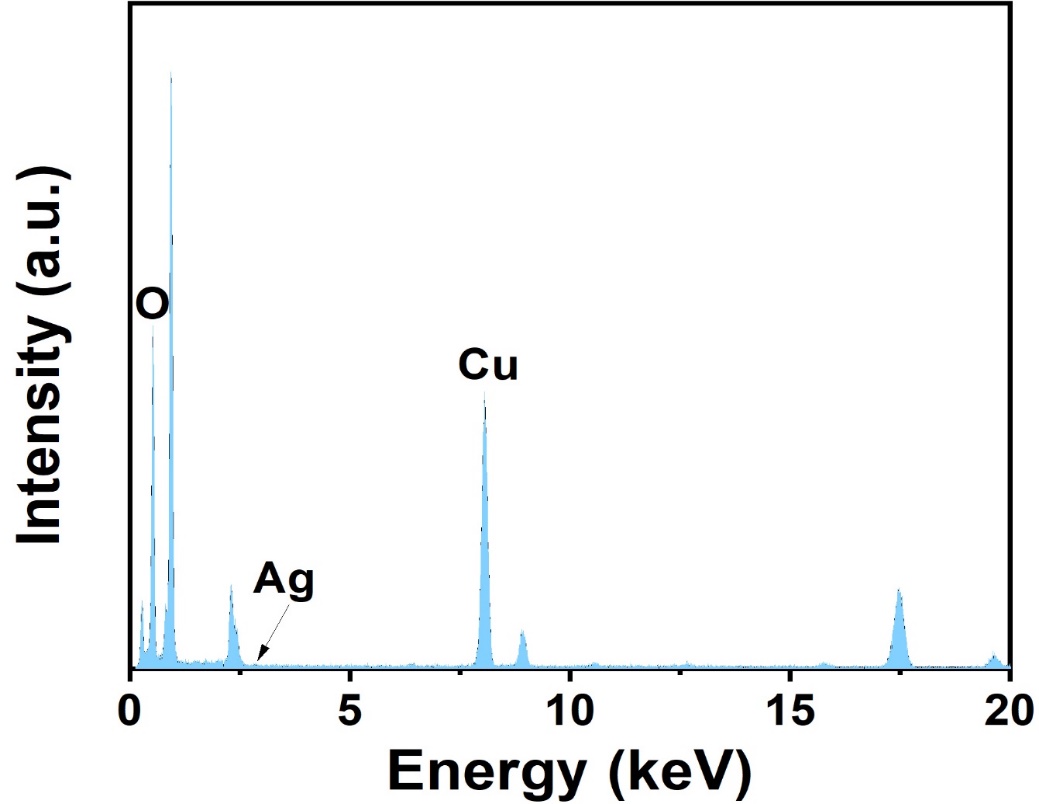


**Figure S1.** EDS spectra of the Ag-CuO nanozymes.


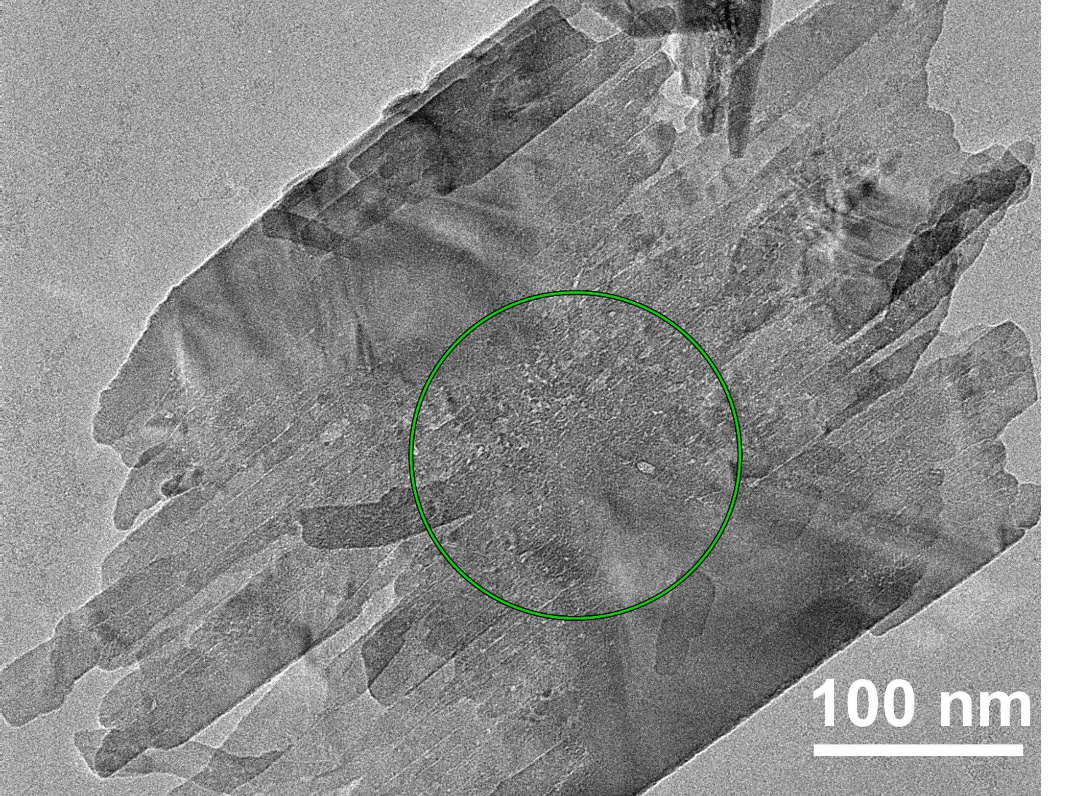


**Figure S2.** Selected area of the electron diffraction pattern of the Ag-CuO nanozymes.


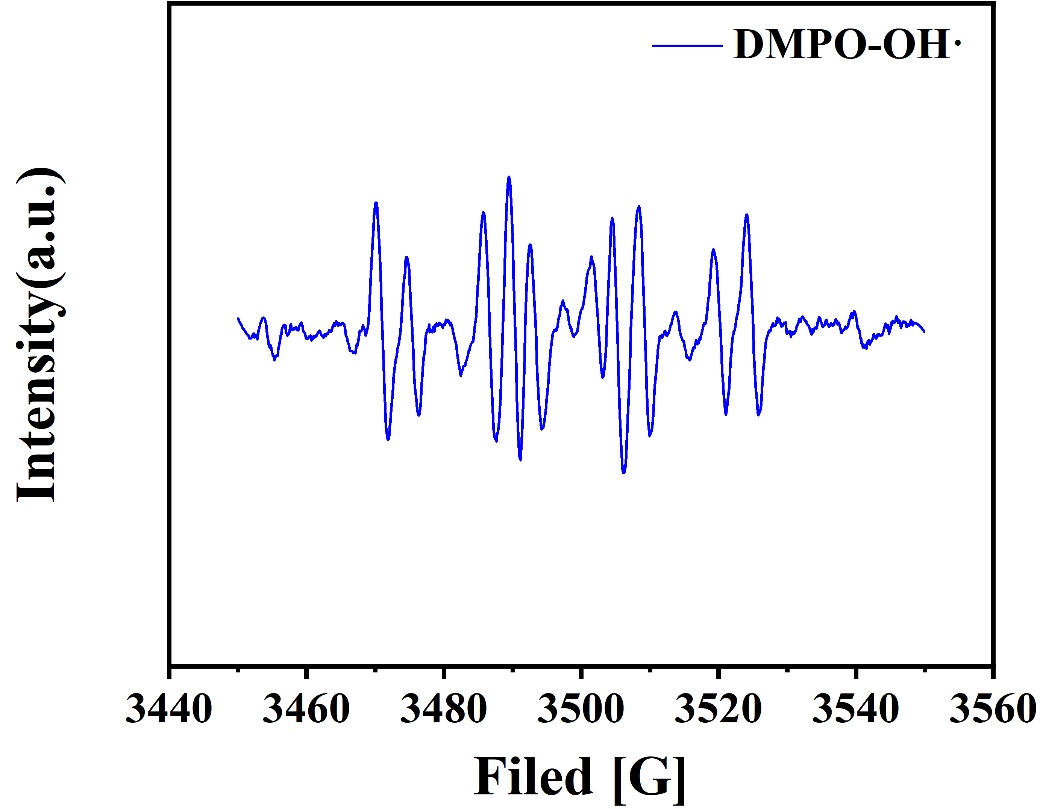


**Figure S3.** Electron paramagnetic resonance (EPR) spectrum of hydroxyl radicals (OH·) generated by Ag-CuO nanozyme-catalyzed H_2_O_2_.


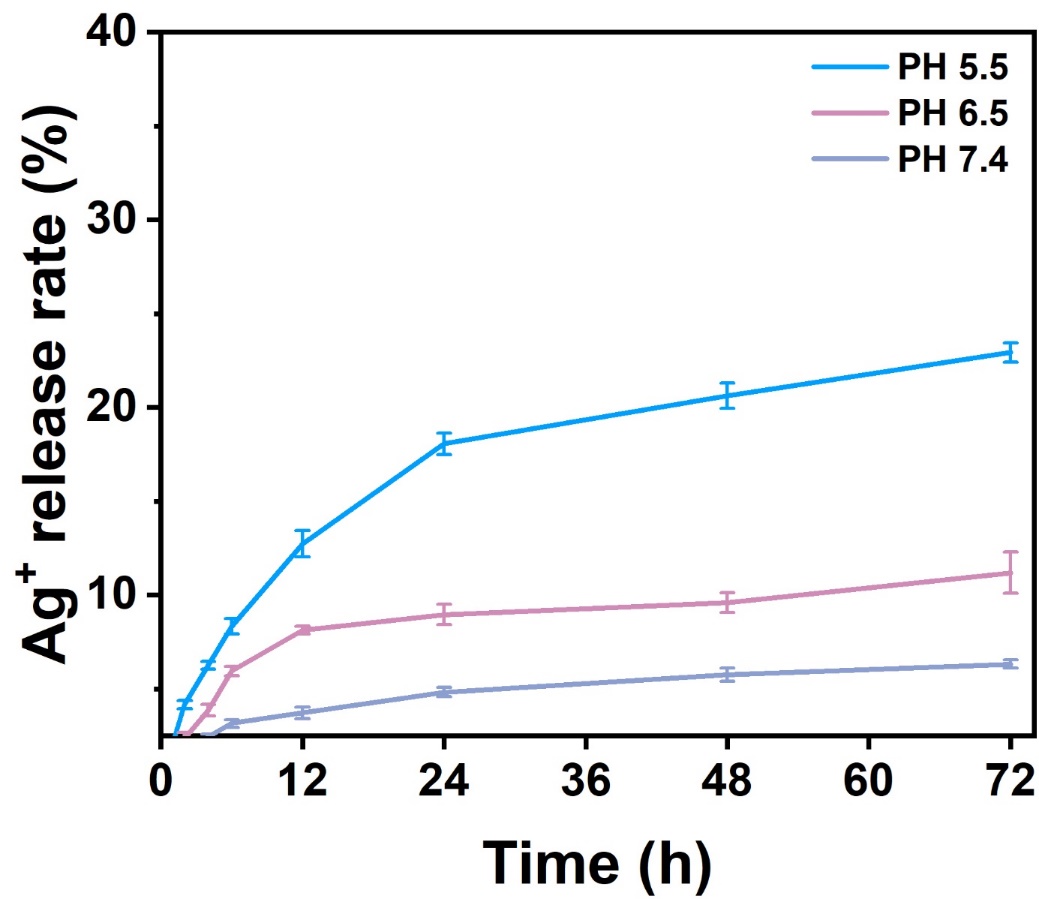


**Figure S4.** Curves of Ag ions released from Ag-CuO nanozymes in different pH solutions (*n* = 3).


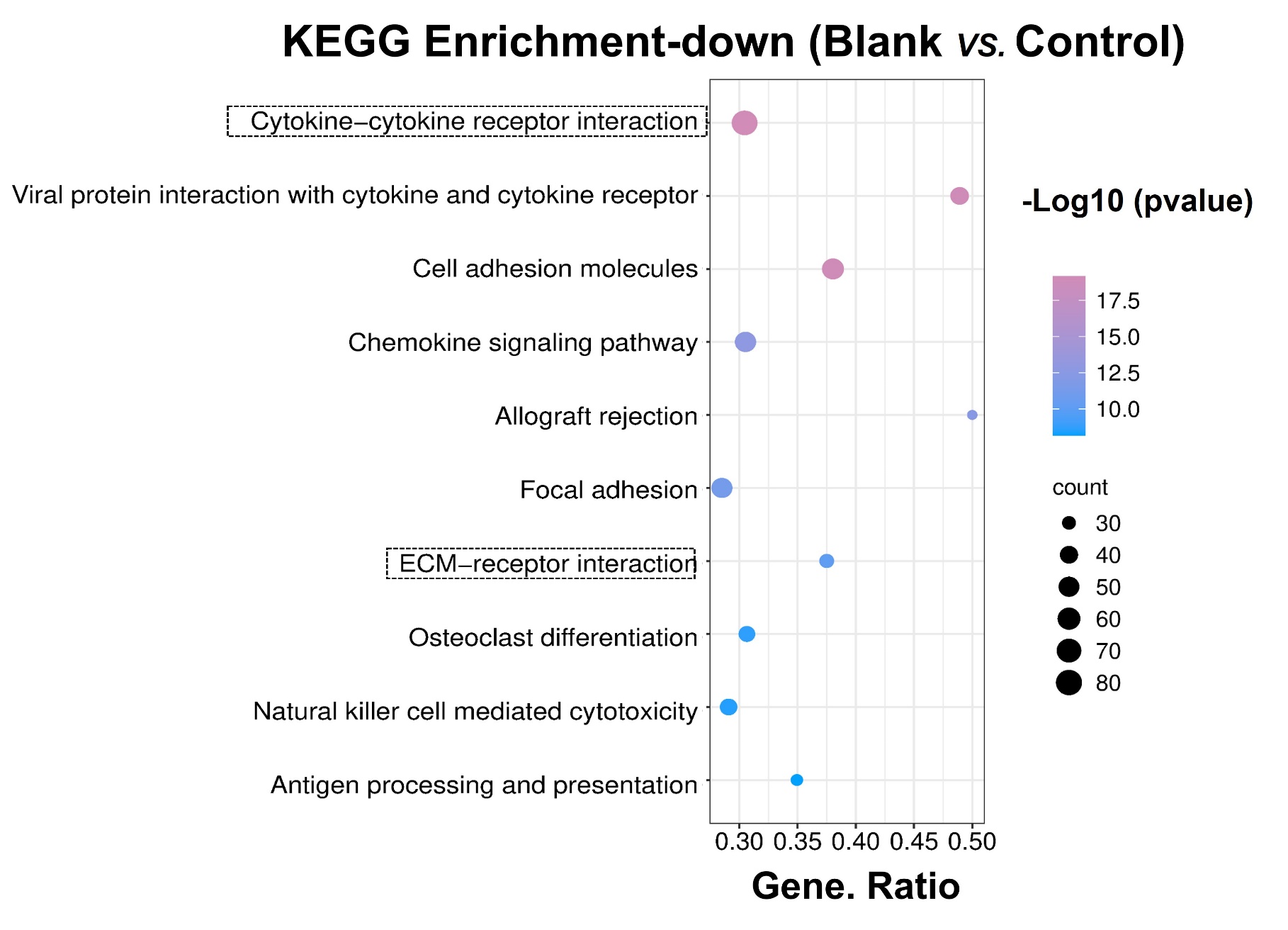


**Figure S5.** The enrichment map of KEGG downregulated pathways (blank vs. control).


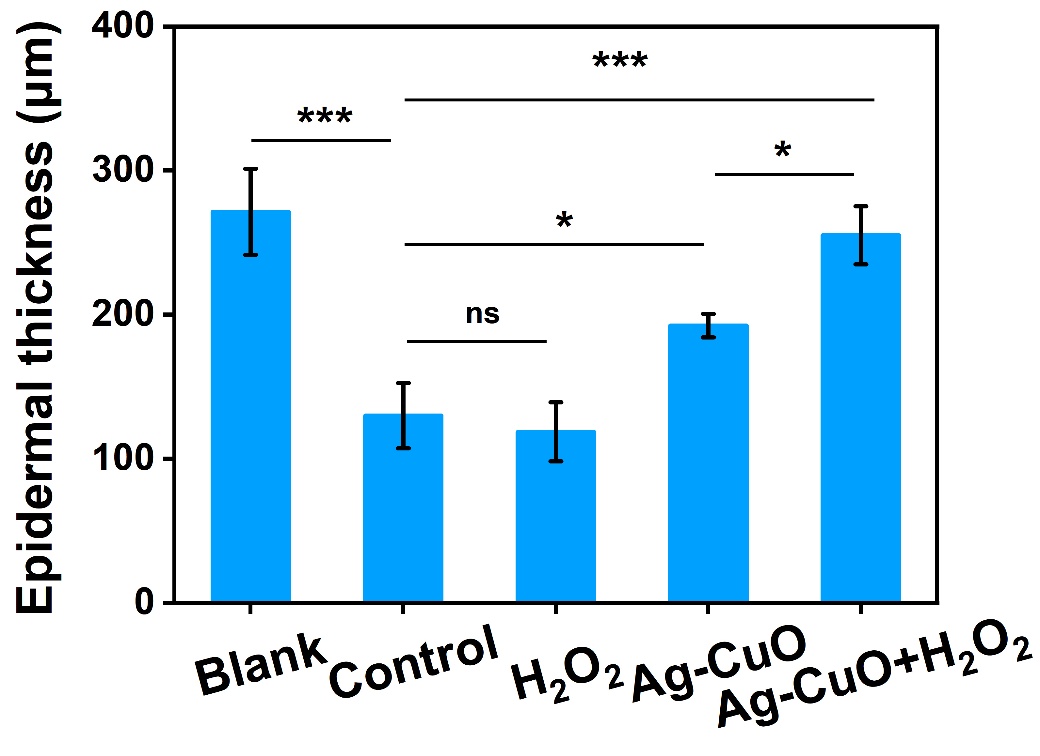


**Figure S6.** Quantiative analysis of epidermal thickness of wounds in different treatment groups. The data are expressed as the means ± SDs (*n* = 3, ***P < 0.001, **P < 0.01, and *P < 0.05).


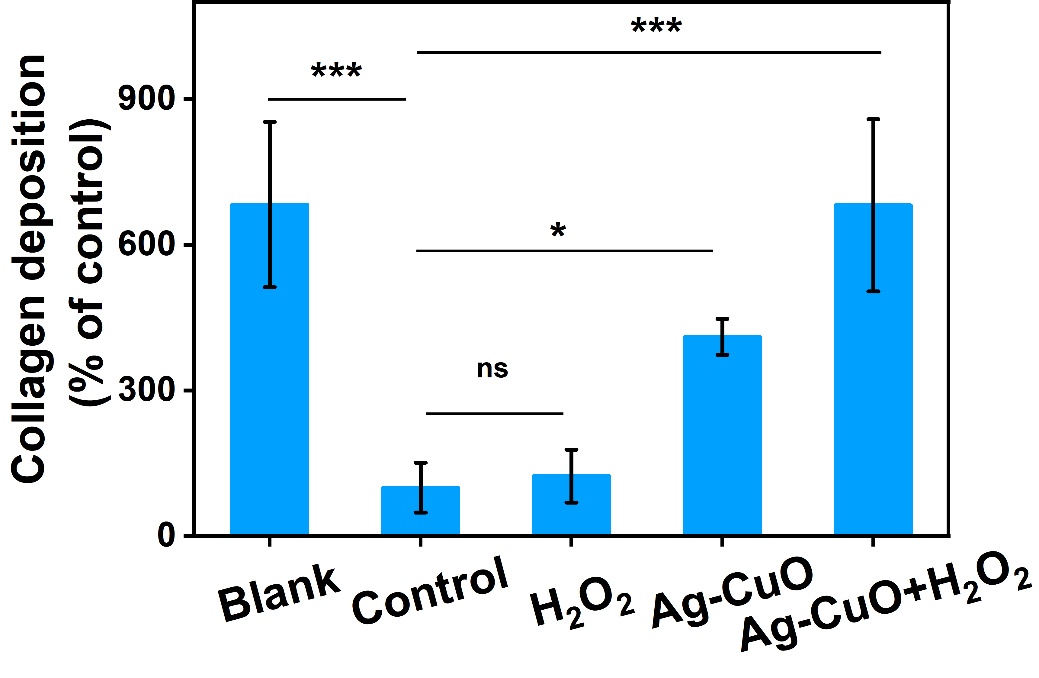


**Figure S7.** Quantiative analysis of collagen deposition of wounds in different treatment groups. The data are expressed as the means ± SDs (*n* = 3, ***P < 0.001, **P < 0.01, and *P < 0.05).


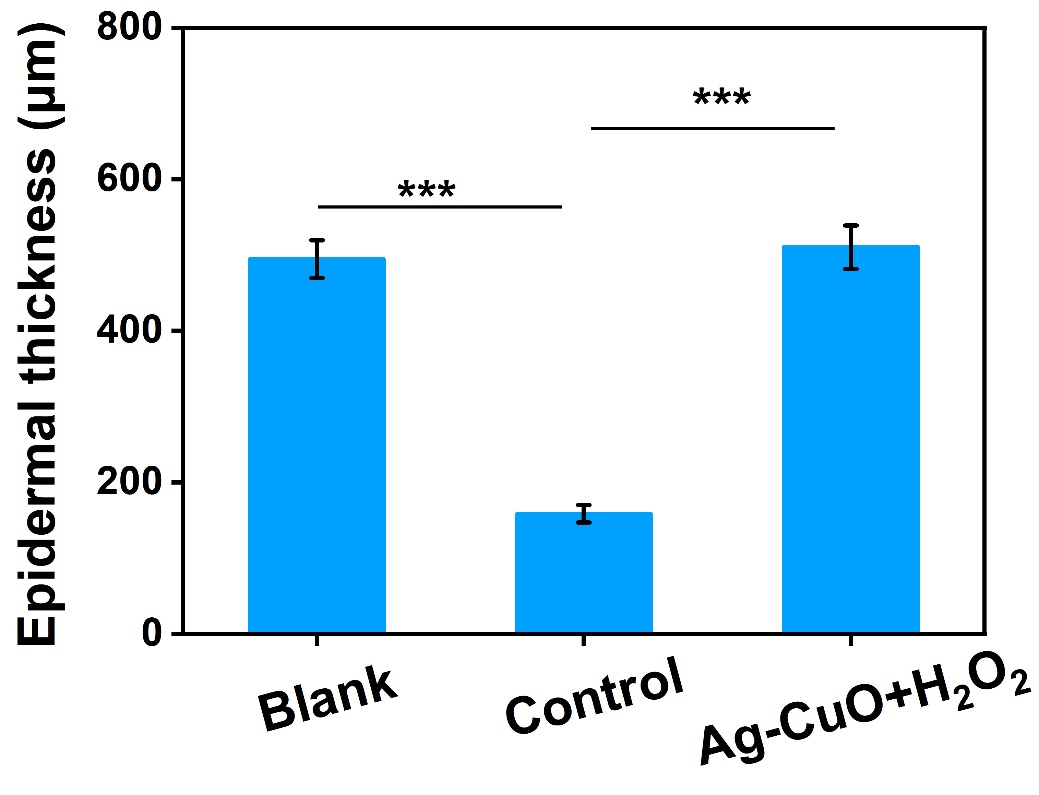


**Figure S8.** Quantiative analysis of epidermal thickness of wounds in different treatment groups. The data are expressed as the means ± SDs (*n* = 3, ***P < 0.001, **P < 0.01, and *P < 0.05).


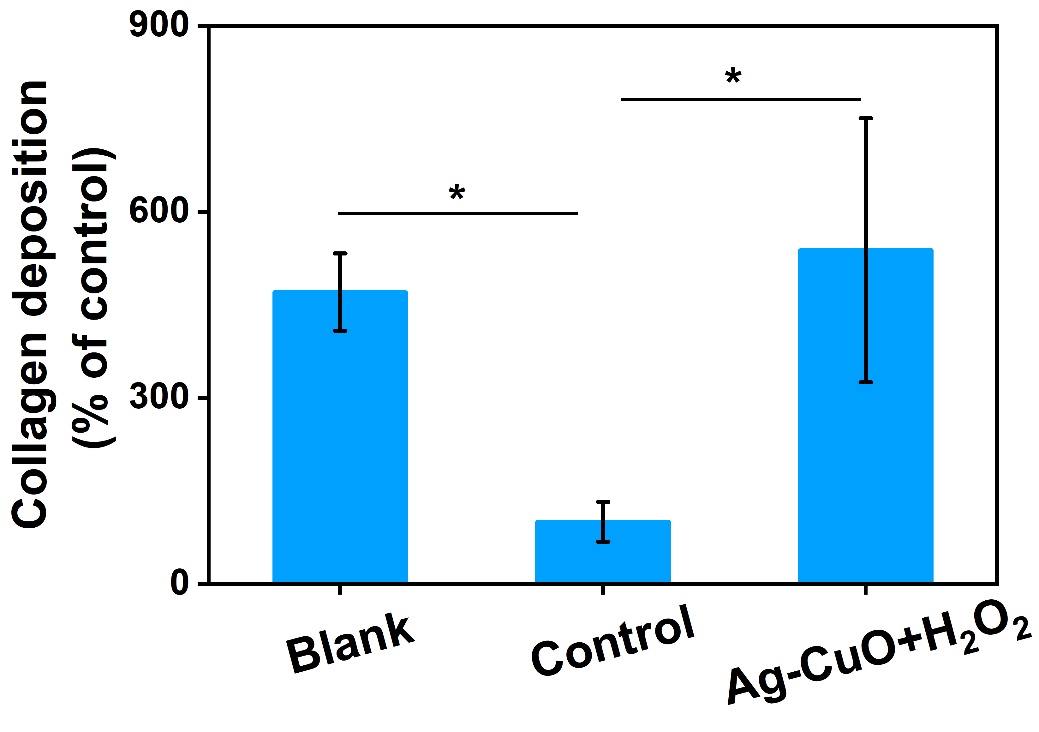


**Figure S9.** Quantiative analysis of collagen deposition of wounds in different treatment groups. The data are expressed as the means ± SDs (*n* = 3, ***P < 0.001, **P < 0.01, and *P < 0.05).


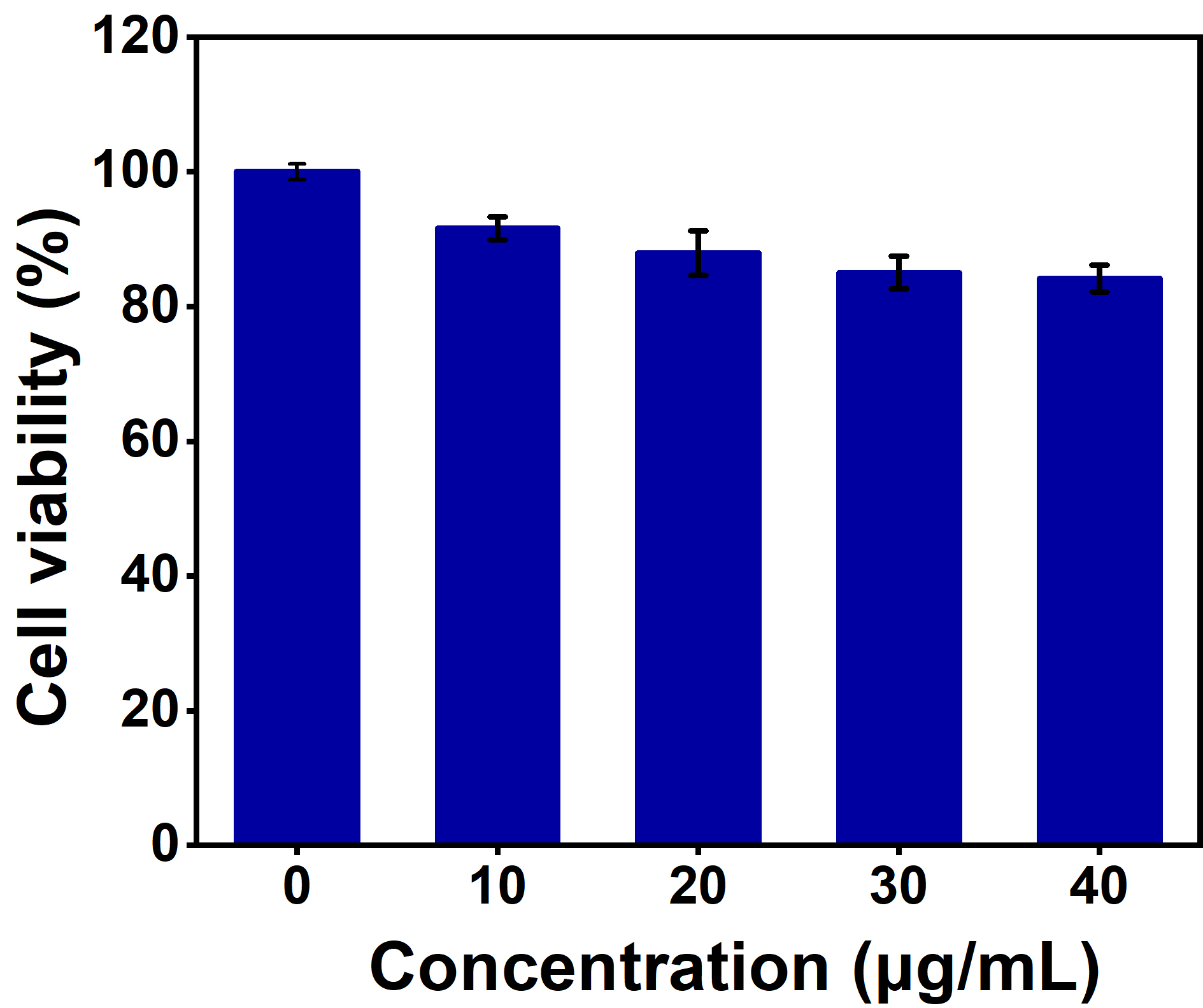


**Figure S10.** Effects of different concentrations of Ag-CuO nanozymes on HUVEC proliferation. The data are presented as the means ± SDs (*n* = 3).

.
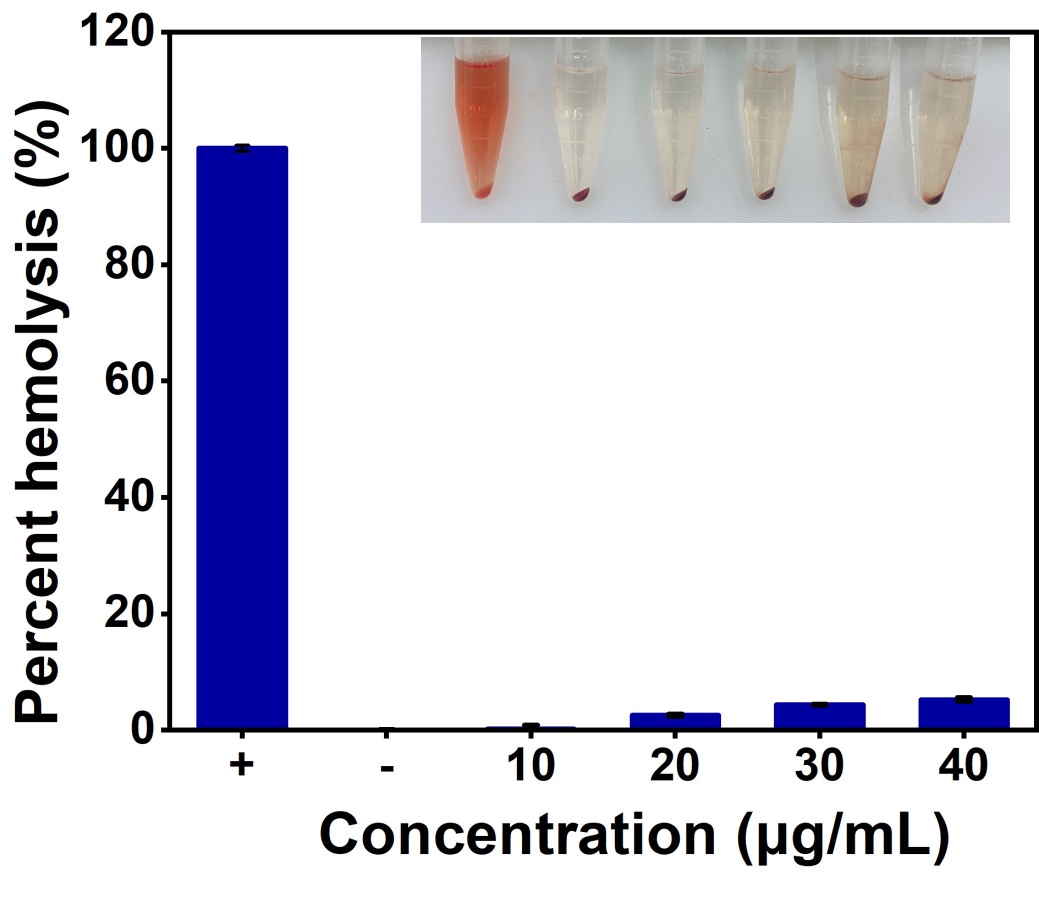


**Figure S11.** Effects of different concentrations of Ag-CuO nanozymes on the hemolysis ratio. The data are presented as the means ± SDs (*n* = 3).


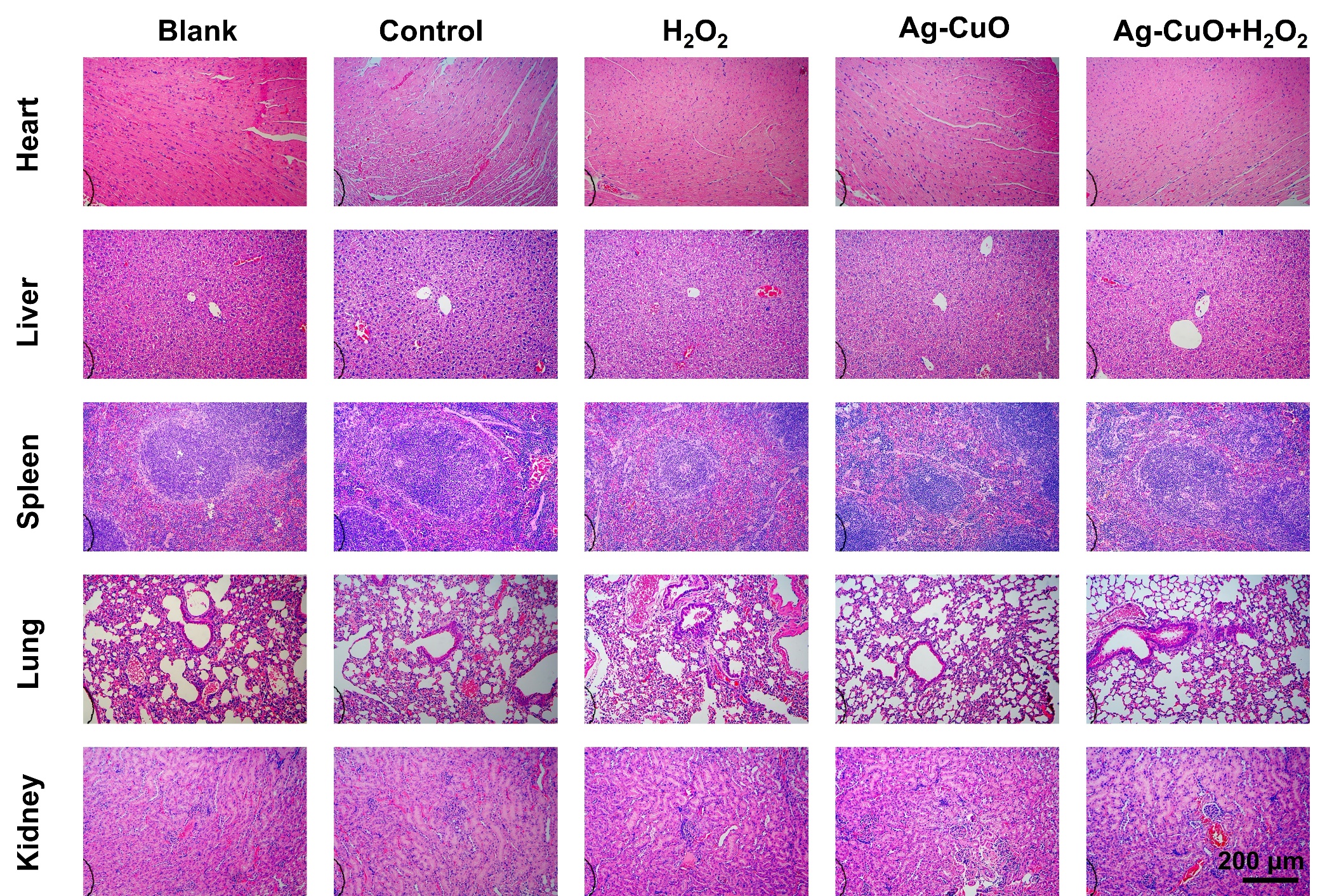


**Figure S12.** H&E staining of major organs (the heart, liver, spleen, lung, and kidney) of mice in different groups on day 14 (n=3).


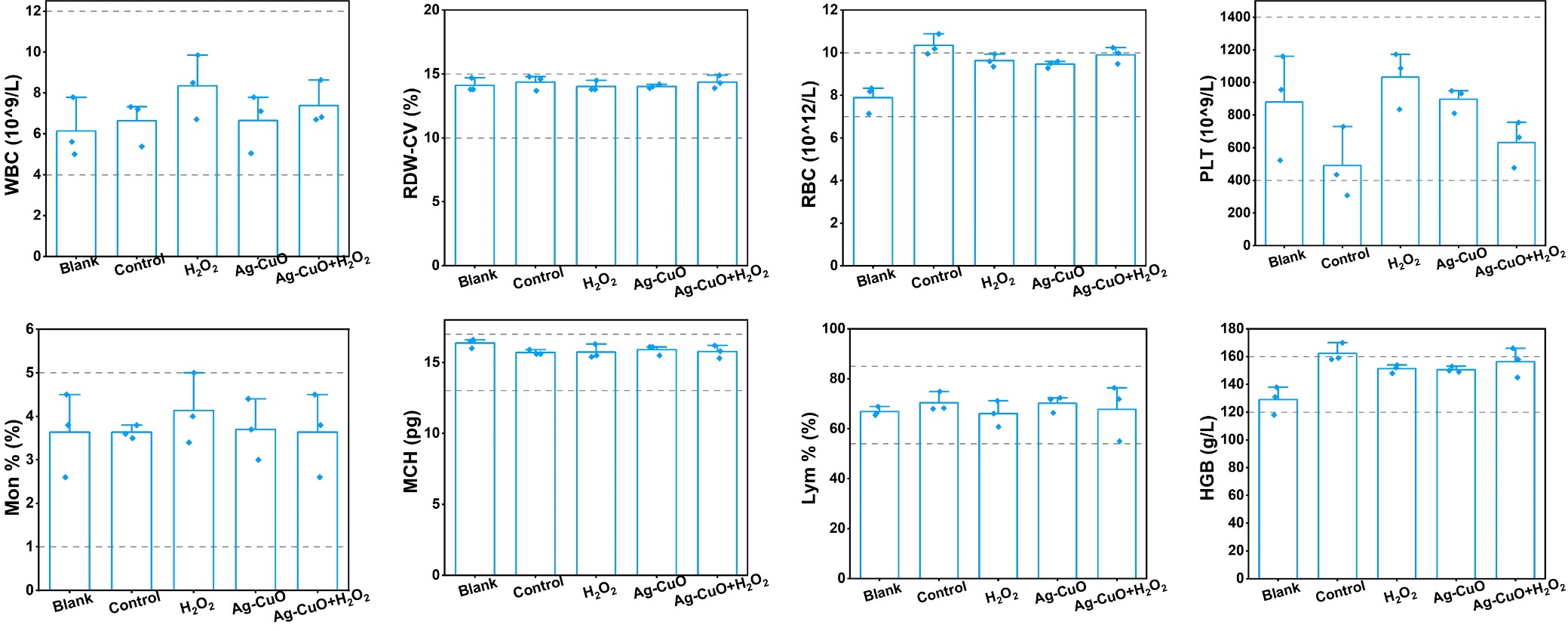


**Figure S13.** Routine blood parameters (including HGB, Lym, MCH, Mon, PLT, RBC, RDW-CV, and WBC) of mice in different groups were measured on day 14 (n = 3).


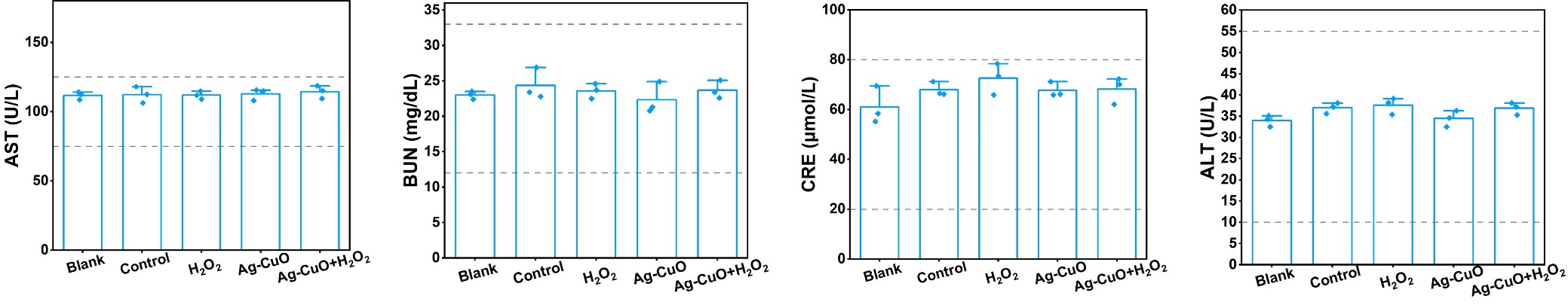


**Figure S14.** Biochemical indices (including ALT, AST, BUN, and CRE) of mice in different groups were measured on day 14 (n = 3).


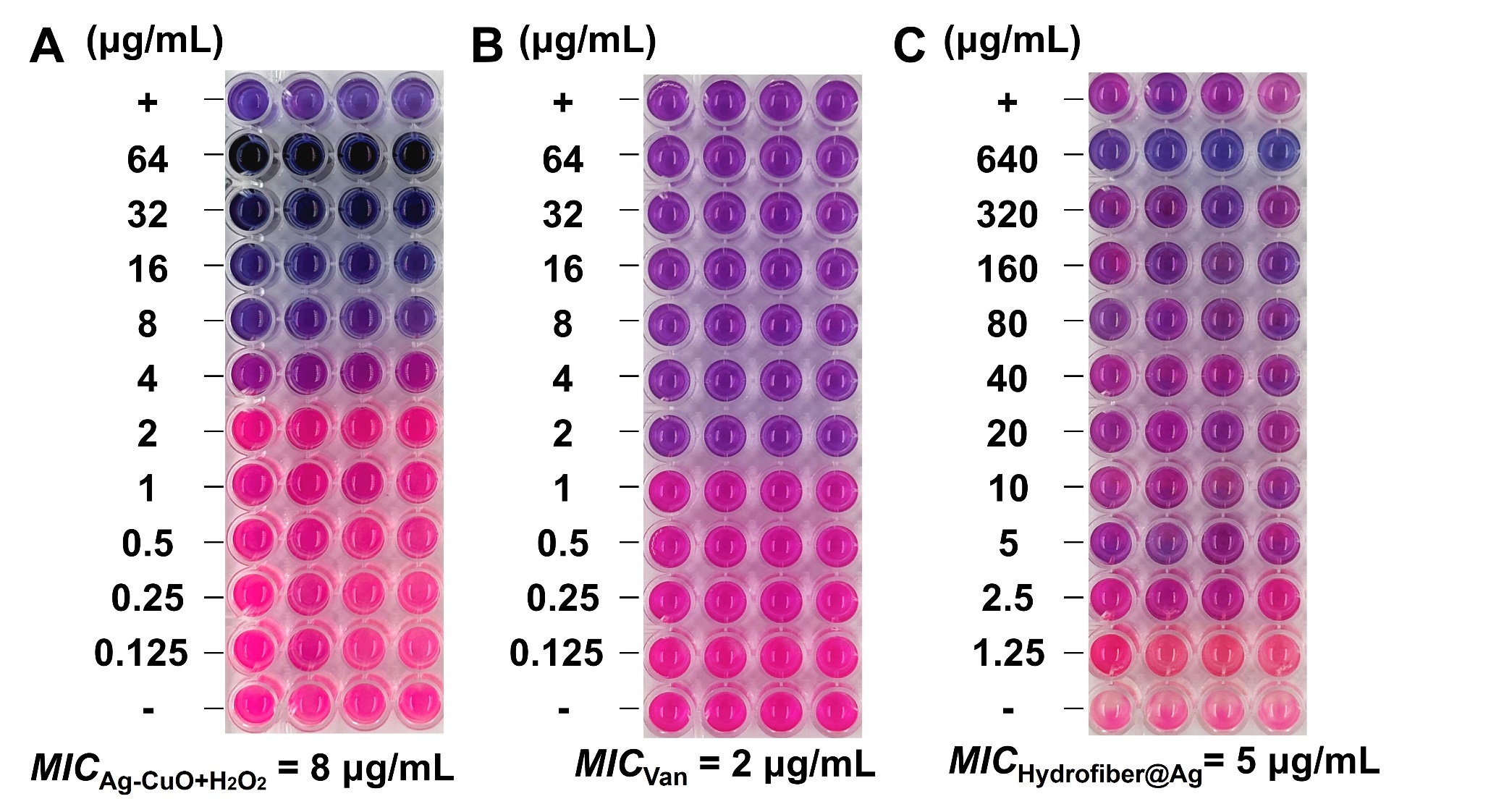


**Figure S15.** MICs of three drugs against *E. faecalis* detected by resazurin staining (n = 4).

**Figure S16.** Effects of different concentrations of Hydrofiber@Ag on HUVEC proliferation. The data are presented as the means ± SDs (*n* = 3).

**Figure S17.** Effects of different concentrations of Van on HUVEC proliferation. The data are presented as the means ± SDs (*n* = 3).


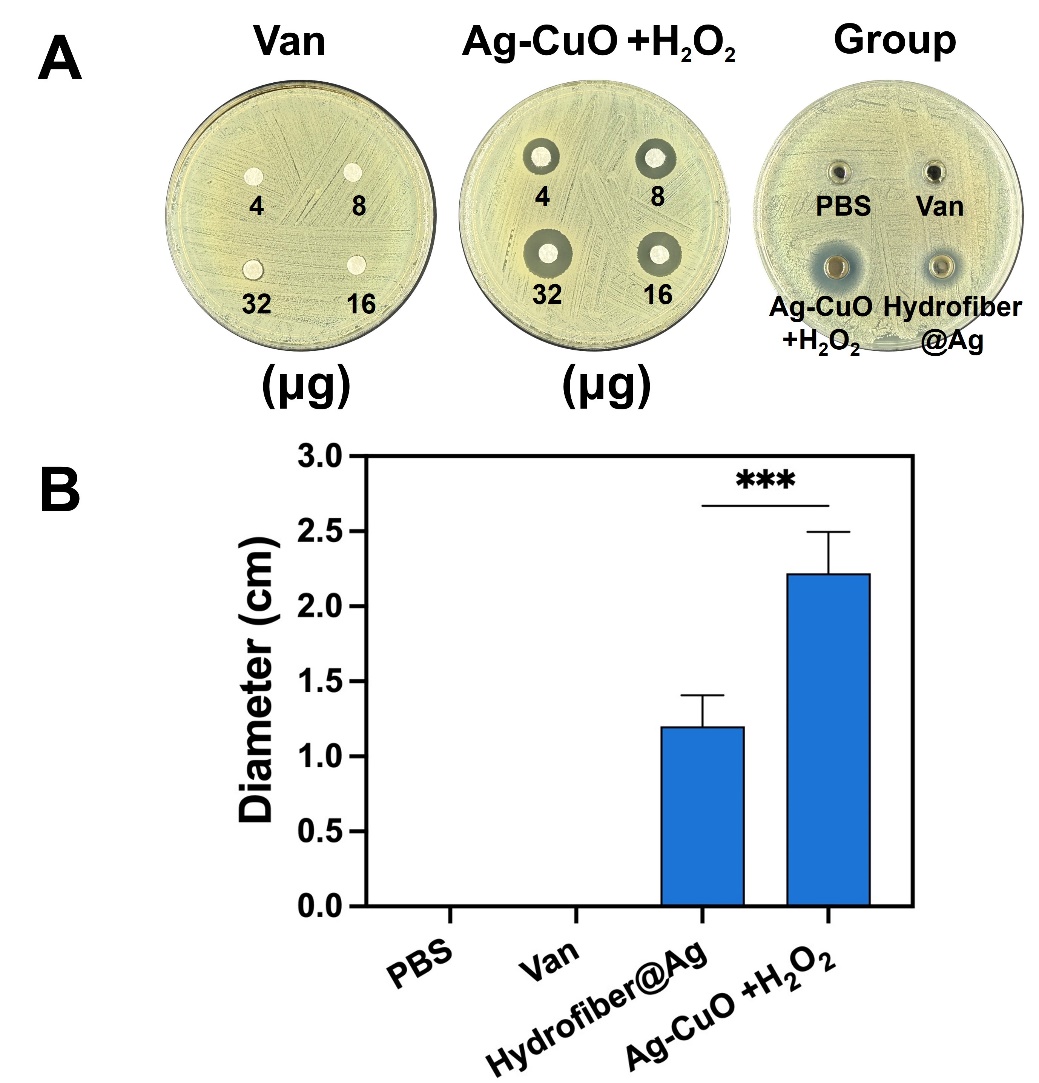


**Figure S18. (A)** Inhibition zone experiments of different concentrations of Van, different concentrations of Ag-CuO nanozymes and different groups of drugs on *VRE* (n = 3). **(B)** Diameter of the inhibition zone of different groups of drugs on *VRE* (n = 3).


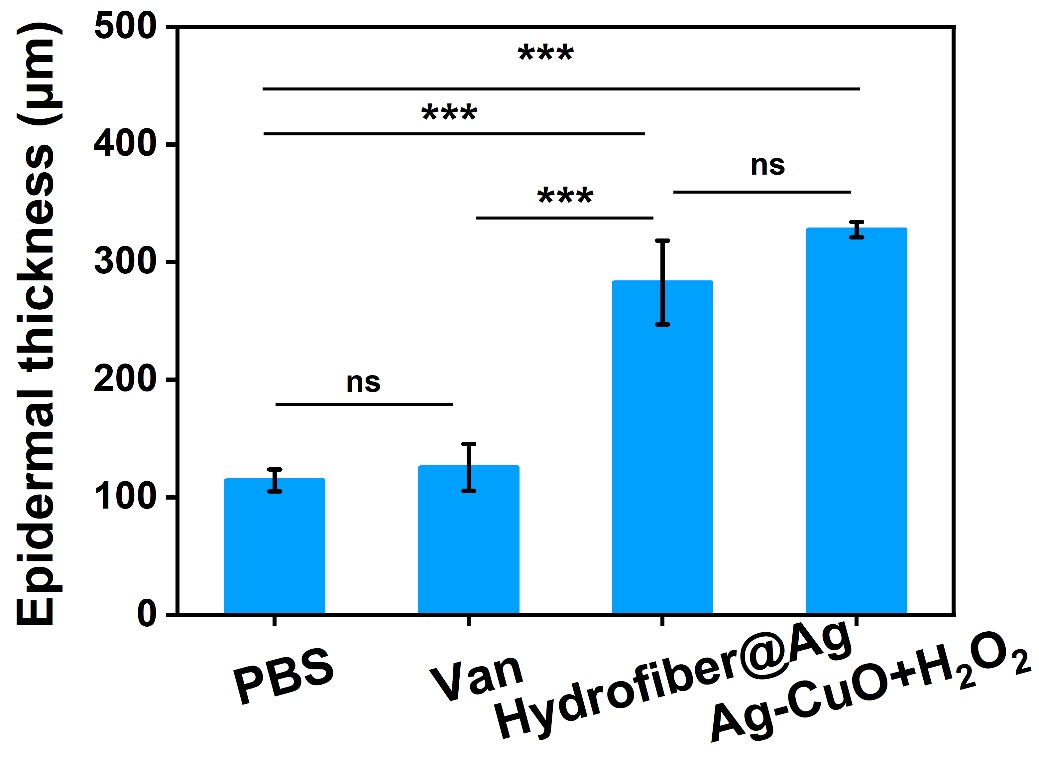


**Figure S19.** Quantitative analysis of the epidermal thickness of wounds in different treatment groups. The data are expressed as the means ± SDs (*n* = 3, ***P < 0.001, **P < 0.01, and *P < 0.05).


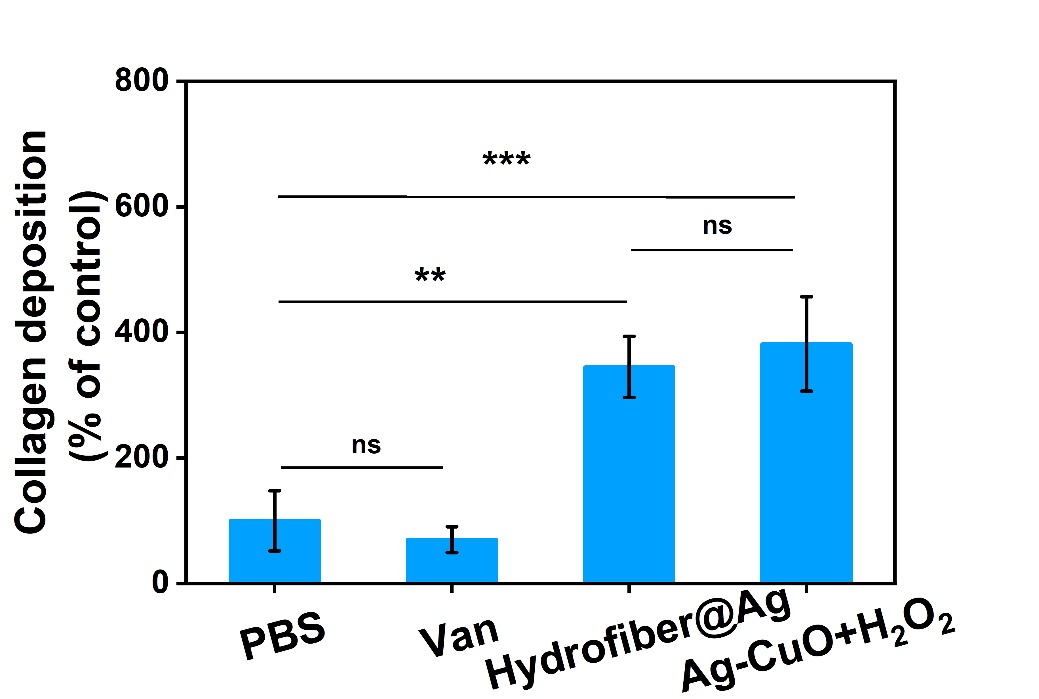


**Figure S20.** Quantitative analysis of collagen deposition in wounds in different treatment groups. The data are expressed as the means ± SDs (*n* = 3, ***P < 0.001, **P < 0.01, and *P < 0.05).


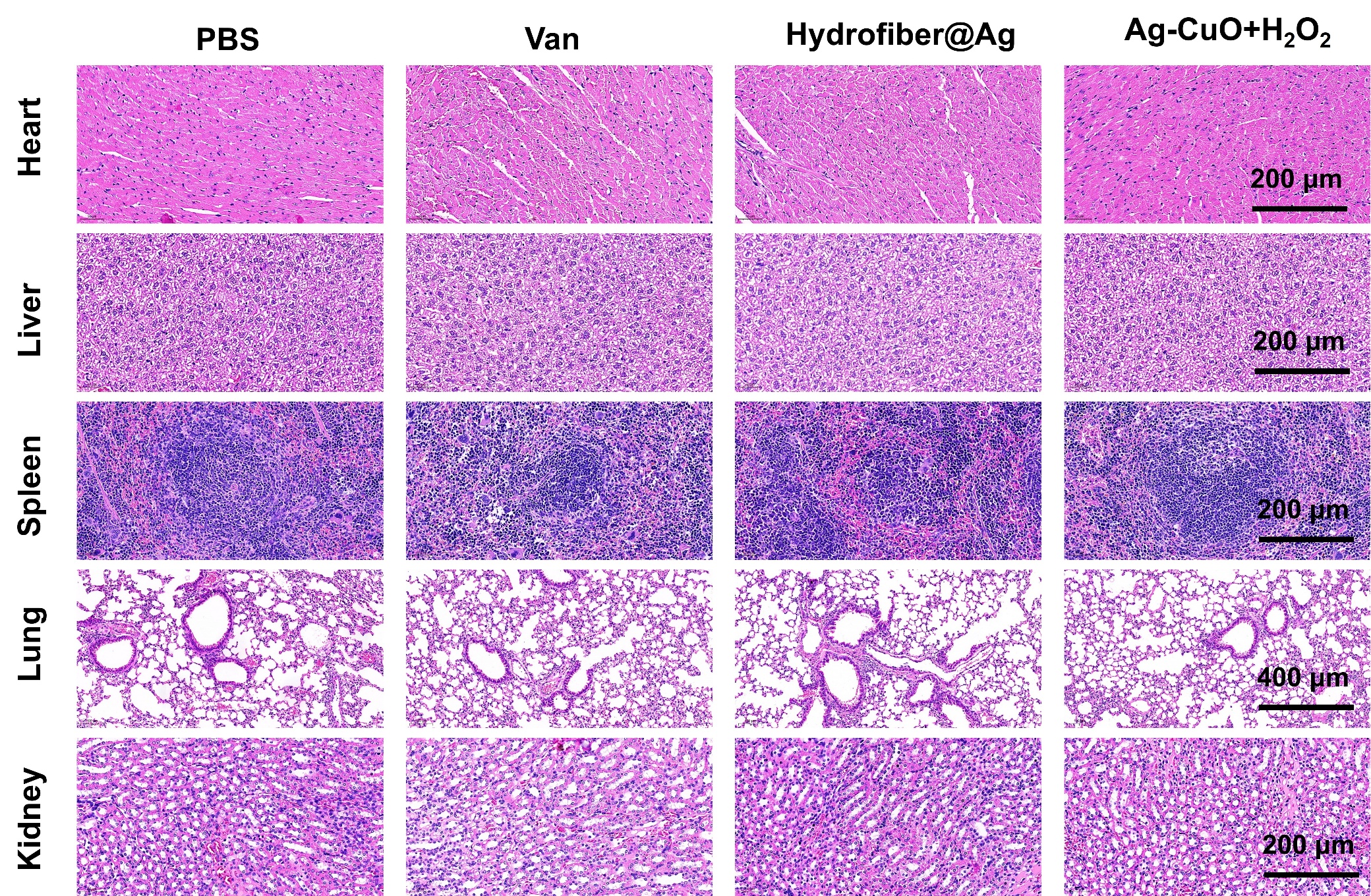


**Figure S21.** H&E staining of major organs (heart, liver, spleen, lung, and kidney) of mice in different groups on day 14 (n=3).
